# Magnetorheological Finishing Technology: Research Progress in Materials, Mechanisms, Equipment, and Intelligentization

**DOI:** 10.3390/mi17070842

**Published:** 2026-07-15

**Authors:** Lingzhi Ding, Guangchao Song, Guili Gao, Dequan Shi

**Affiliations:** 1 School of Materials and Chemistry, University of Shanghai for Science and Technology, Shanghai 200093, China; 255393152@st.usst.edu.cn; 2 School of Materials Science and Engineering, Shanghai Changxing Ocean Laboratory, Shanghai Jiao Tong University, Shanghai 200240, China; songgua1@sjtu.edu.cn; 3 School of Mechanical and Energy Engineering, Shanghai Technical Institute of Electronics and Information, Shanghai 201411, China; gao-guili@163.com

**Keywords:** magnetorheological finishing, ultra-precision machining, material removal mechanism, polishing fluid, hybrid polishing technology, intelligent manufacturing

## Abstract

Magnetorheological Finishing (MRF) technology, as a deterministic machining method featuring flexibility, controllability, and extremely low subsurface damage, has become one of the most promising technologies in the field of ultra-precision polishing. This review systematically summarizes the research progress of MRF technology in aspects such as polishing fluid preparation and rheological properties, material removal mechanisms and theoretical models, classification and optimization of equipment and processes, multi-energy field hybrid technologies, and intelligent development. First, starting from the composition, nonlinear rheological models, and stability optimization of the magnetorheological polishing fluid, the decisive influence of fluid properties on finishing performance is elucidated. Second, the macroscopic mechanical removal mechanism and the atomic-scale material removal mechanism are analyzed, and the establishment and development of material removal function and polishing force models are reviewed. Third, MRF equipment is systematically classified according to tool morphology, and advanced hybrid polishing technologies such as ultrasonic-assisted, electrochemical-assisted, and laser-assisted methods are discussed. Furthermore, the effects of key process parameters, magnetic field configuration optimization, and dwell time algorithms on machining accuracy and efficiency are analyzed in depth. Finally, typical applications of MRF technology in aerospace, biomedical, optoelectronic information and other fields are summarized, the current technical bottlenecks are pointed out, and future development trends toward intelligence, greenization, and standardization are prospected. This review aims to provide comprehensive theoretical references and technical guidance for researchers in the MRF field, and to promote further application and development of this technology in precision manufacturing.

## 1. Introduction

### 1.1. Research Background and Significance

The rapid development of modern precision manufacturing has placed unprecedented high demands on the surface quality of components. In fields such as aerospace, optical observation, semiconductor manufacturing, and biomedical engineering, many core components require nanoscale or even sub-nanoscale surface roughness, while also demanding extremely low subsurface damage and micrometer-scale form accuracy [1,2,3,4]. For example, large-aperture optical mirrors used in deep space exploration require a surface roughness (Ra) below 1 nm; KDP crystals used in high-power laser fusion facilities demand of an extremely high laser-induced damage threshold; and titanium alloy implants for artificial joints need ultra-smooth surfaces to ensure biocompatibility and wear resistance [5,6,7].

Traditional ultra-precision polishing technologies mainly include Chemical Mechanical Polishing (CMP), Ion Beam Figuring (IBF), Elastic Emission Machining (EEM), and Abrasive Flow Machining (AFM), among others [8,9,10]. Although CMP can achieve nanoscale surface roughness, it typically uses polishing fluids containing toxic chemical reagents, which poses environmental pollution problems and tends to introduce residual stress on the material surface [11]. Non-contact processing methods such as IBF and EEM can avoid mechanical damage, but they suffer from high equipment costs, extremely low processing efficiency, and difficulty in achieving large-scale production [12]. Although AFM can process complex internal channels, its material removal rate is relatively low, making it difficult to meet the demands of mass production [13].

**Figure 1:**
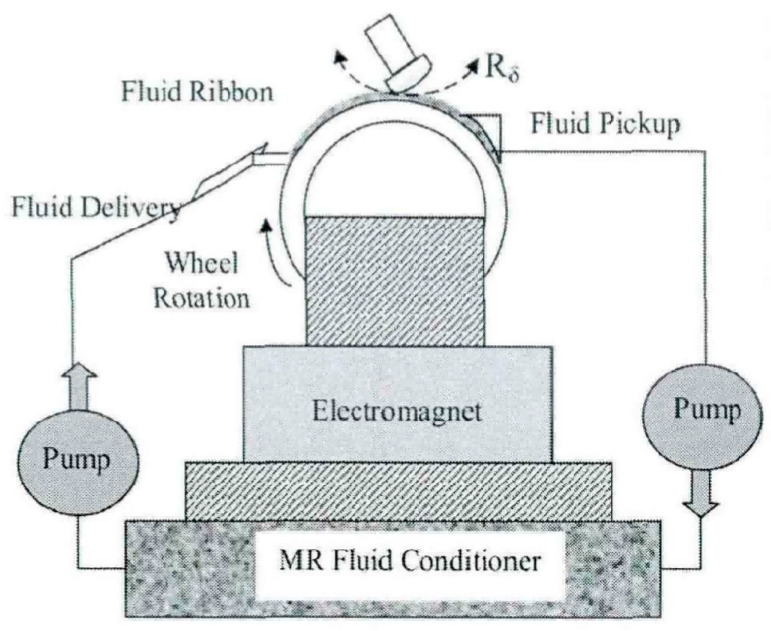
Schematic of magnetorheological finishing [16].

The emergence of Magnetorheological Finishing (MRF) technology (Figure 1) provides an effective approach to addressing the aforementioned bottlenecks. MRF technology utilizes the instantaneous rheological effect of magnetorheological fluid in a gradient magnetic field to form a viscoplastic flexible polishing film. By controlling the magnetic field intensity, the polishing pressure and shear force can be precisely adjusted, thereby achieving deterministic material removal on the workpiece surface [14,15,16]. This flexible-contact processing method introduces no subsurface damage and enables instant renewal of the polishing layer, ensuring the stability and consistency of the process. Furthermore, MRF technology possesses the dual functions of surface smoothing and form correction, and can process a wide range of materials from optical glass, crystals, and ceramics to metal alloys, covering various complex surface shapes including planes, spheres, aspheres, and freeform surfaces. For example, to address the polishing challenges of internal surfaces of titanium alloy tubes for aerospace applications, Song et al. developed a novel magnetorheological finishing (MRF) device with a compound magnetic field. By employing a combined motion of tube rotation and reciprocating movement of the polishing head, along with hybrid permanent-magnet and electromagnet excitation, the study systematically optimized magnetic field intensity, rotational speed, and abrasive parameters. As a result, they successfully achieved micron-level polishing of the internal surfaces of titanium alloy tubes, demonstrating the great potential of MRF technology in solving polishing problems for specific difficult-to-machine materials and workpieces with complex geometries [17,18,19].

With the deepening advancement of industrial and intelligent manufacturing concepts, MRF technology is rapidly developing toward intelligence, hybridization, and greenization. In recent years, numerous research efforts have been conducted by scholars worldwide on MRF technology, achieving significant progress in polishing fluid development, material removal mechanisms, equipment development, process optimization, and hybrid processing. However, MRF technology still faces challenges such as the trade-off between processing efficiency and accuracy, difficulty in suppressing edge effects, and limited recycling service life of polishing fluid.

### 1.2. Development History of Magnetorheological Finishing Technology

The development of magnetorheological finishing technology can be traced back to the late 1940s. In 1948, Rabinow first discovered the magnetorheological phenomenon: magnetic fluids exhibit good fluidity under a zero magnetic field but display solid-like characteristics under a strong magnetic field. This continuous and controllable transition phenomenon was later termed the magnetorheological effect [20]. However, due to insufficient understanding of the rheological properties of magnetorheological fluids at that time, the development of this technology progressed slowly for more than three decades thereafter.

In the 1970s, Kordonski’s team first applied magnetorheological fluid to the field of mechanical processing, pioneering magnetorheological finishing technology [21]. In 1986, Kordonski and colleagues confirmed that a properly formulated magnetorheological fluid could be used to polish optical materials under a magnetic field, marking the official birth of MRF technology. In 1992, Kordonski designed the world’s first prototype magnetorheological finishing machine. Although this equipment did not yet incorporate the concept of Computer Controlled Optical Surfacing (CCOS) and could only process aspheric surfaces without achieving high-precision form correction, it laid an important foundation for subsequent technological development [22].

In the 1990s, with the vigorous development of the medical device, optoelectronics, and communication industries, the pursuit of enhanced product performance, reliability, and quality strongly promoted the rapid advancement of MRF technology. In 1993, Kordonski collaborated with the Center for Optics Manufacturing (COM) at the University of Rochester in the United States to develop a new-generation prototype magnetorheological finishing machine. Through extensive experiments, they validated the advantages of MRF technology for efficient, low-damage processing of optical materials [23]. In 1995, COM developed the first orthogonal wheel-type magnetorheological finishing equipment, successfully processing high-quality optical components with a surface roughness (Ra) of 1 nm, ushering in an era of innovation for MRF technology [15].

The year 1996 marked an important turning point in the history of MRF technology. COM integrated the CCOS concept into the wheel-type polishing equipment, achieving computer numerical control (CNC) machining and ushering MRF technology into the era of intelligence. In 1997, COM established QED Company, beginning the commercialization of magnetorheological finishing equipment [24]. Since then, QED has rapidly grown into the global leader in magnetorheological finishing and related technologies, developing a series of mature commercial equipment ranging from the Q22-400 to the Q22-2000F. These systems are equipped with polishing wheels whose diameters vary from 10 mm to 370 mm, and are capable of processing concave mirrors up to 2.3 m and convex mirrors up to 1.7 m.

**Figure 2:**
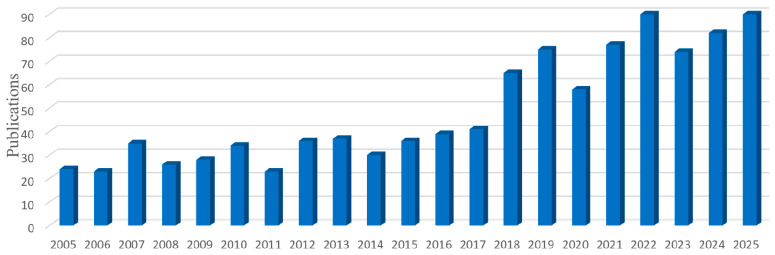
Number of publications on magnetorheological polishing technology from 2005 to 2025.

Since the beginning of the 21st century, research on MRF technology has experienced explosive growth worldwide. According to statistics from the Web of Science database, the annual number of publications using “magnetorheological finishing” as a keyword has risen from fewer than 10 in 2001 to nearly 100 in recent years [7] (Figure 2). The research content has also expanded from the initial equipment development to diversified directions such as polishing fluid formulation optimization, rheological theory modeling, material removal function analysis, trajectory planning and dwell time algorithms, and multi-energy field hybrid processing [25,26,27,28].

China started relatively late in the field of MRF technology research but has developed rapidly. Previous studies have reported that many universities and research institutions have accumulated rich experience in wheel-type polishing, ball-end polishing, cluster polishing, and hybrid MRF [29,30,31,32,33].

### 1.3. Purpose, Scope, and Structure of the Review

Although several reviews have summarized specific aspects of MRF technology—such as the classification review by Beidi et al. [34] on MRF methods applicable to different surface shapes, the review by Ajay [35] on polishing fluid composition and polishing force, the topical review by Xiao et al. [36] on hybrid MRF technologies, and the review by Jain et al. [37] on magnetorheological abrasive flow finishing—most published reviews focus on a single technical aspect and lack a systematic synthesis from materials, theory, equipment, and processes to applications. Therefore, this review aims to provide a comprehensive analysis of MRF technology, systematically examining its research progress across five dimensions: core medium, fundamental theory, equipment and processes, hybrid technologies, and applications and future trends, thereby offering researchers an overall reference framework.

The organization of this paper is as follows: Section 1 briefly describes the background significance, development history, and research status of MRF technology; Section 2 systematically elaborates on the composition, rheological properties, and development directions of high-performance magnetorheological polishing fluids; Section 3 deeply discusses the material removal mechanisms and theoretical models of MRF; Section 4 introduces typical and advanced hybrid MRF equipment and processes according to tool morphology classification; Section 5 analyzes the research progress of key process parameters, magnetic field optimization, and dwell time algorithms; Section 6 summarizes the typical application fields and current technical challenges of MRF technology; Section 7 prospects the future development trends of MRF technology; and finally, Section 8 provides a conclusion.

To clearly distinguish this review from previous overviews, Table 1 compares the scope, classification method, intelligentization coverage, and process modeling discussion among representative earlier reviews and the present work. Unlike prior reviews that focus on either polishing fluid rheology (e.g., Sidpara), hybrid techniques (e.g., Xiao), or single tool types (e.g., Bedi), the present review integrates all key facets—materials, mechanisms, equipment, hybrid technologies, and intelligent manufacturing—into a unified framework. Specifically, we provide a comprehensive treatment of atomic-scale removal mechanisms via MD simulations, a systematic classification of MRF tools with quantitative performance tables, and an in-depth discussion on machine-learning-assisted process optimization, which are largely absent in previous reviews.

## 2. Magnetorheological Polishing Fluid

The magnetorheological polishing fluid is a core component of MRF technology, and its performance directly determines polishing efficiency, surface quality, and process stability. As a functional suspension, MR polishing fluid is mainly composed of a carrier liquid, magnetic particles, abrasive particles, and functional additives such as surfactants. When subjected to an external magnetic field, the magnetic particles rapidly align along the magnetic field lines to form chain-like structures, entrapping the abrasive particles and transforming the fluid from a Newtonian fluid into a viscoplastic Bingham body with a yield stress [38]. Research on polishing fluid involves multiple aspects, including component optimization, rheological characterization, improvement of sedimentation stability, and the development of novel high-performance polishing fluids.

### 2.1. Composition, Formulation, and Rheological Properties

#### 2.1.1. Core Components

MR polishing fluid is a composite suspension system formed by mixing multiple components in specific proportions. The properties and ratios of each component determine the overall performance of the polishing fluid.

(1) Magnetic particles serve as the skeleton component of MR polishing fluid. Their directional alignment and chain formation under an external magnetic field constitute the physical basis for the magnetorheological effect. The most widely used magnetic particles are carbonyl iron powder (CIP), owing to their soft magnetic properties, high saturation magnetization, and good dispersibility [39]. The diameter of CIP typically ranges from 1 to 10 μm, with a volume fraction generally between 20% and 40%. Studies have shown that the particle size, morphology, and concentration of CIP significantly affect the rheological properties of MR polishing fluid. Ginder and Davis [40] found that increasing CIP particle size within a certain range can significantly enhance the shear yield stress, but beyond a critical size, the yield stress tends to stabilize. Upadhyay et al. [41] demonstrated that, compared with spherical particles, rod-shaped particles can form stronger chain-like structures under oscillatory shear, thereby enhancing the magnetorheological effect. Lee et al. [42] observed that plate-shaped particles can reduce the sedimentation rate by nearly 50%.

To improve dispersibility and oxidation resistance, researchers often apply surface modification treatments to CIP. Common modification methods include coating the surface of CIP with organic or inorganic coatings, such as oleic acid, polyvinyl alcohol, and silicon dioxide [43,44,45]. Machovsky et al. [46] prepared ZnO-coated CIP, which not only improved oxidation resistance and mechanical strength but also achieved a yield stress of 2.2 kPa under a magnetic field of 272 mT and a concentration of 60 wt%. In recent years, the development of core–shell structured magnetic particles (such as CI/PMMA, CI/γ-Fe_2_O_3_, CI/polyaniline-multiwalled carbon nanotubes, etc.) has become a research hotspot. These composite particles suppress sedimentation by reducing their effective density while retaining good magnetic responsiveness [47,48,49].

(2) The carrier liquid provides a suspension medium for the magnetic particles and abrasive particles, and endows the polishing fluid with basic fluidity. According to the type of carrier liquid, MR polishing fluids can be classified into two major categories: water-based and oil-based [50]. Water-based MR polishing fluid offering advantages such as low viscosity, high magnetic response speed, good cooling performance, and biodegradability. It is also low in cost, non-toxic, and environmentally friendly, making it suitable for polishing most water-insoluble materials [51]. However, water evaporates relatively quickly, which can easily cause changes in component proportions during prolonged processing. Oil-based MR polishing fluid possesses excellent lubricity and thermal stability, can meet the processing requirements under harsh conditions such as high temperature and high pressure, and can effectively inhibit the oxidation and sedimentation of magnetic particles [23,52]. Its disadvantages include poor biodegradability, environmental unfriendliness, and relatively high cost. In practical applications, a comprehensive selection should be made based on factors such as the characteristics of the workpiece material, processing efficiency, and environmental requirements.

(3) Abrasive particles serve as the active units that achieve material removal from the workpiece surface. Their hardness, size, shape, and concentration have a decisive influence on the material removal rate (MRR) and the resulting surface quality. Commonly used abrasives include silicon carbide (SiC), aluminum oxide (Al_2_O_3_), cerium oxide (CeO_2_), boron carbide (B_4_C), diamond powder, and composite abrasives [53]. Among the commonly used abrasives, diamond exhibits the highest hardness, enabling efficient material removal for hard ceramics but risking surface scratching if not finely controlled. SiC offers a moderate cost-to-performance balance, suitable for semi-finishing of glass and metals. CeO_2_ is significantly softer but possesses high chemical reactivity with SiO_2_-based glasses, facilitating chemomechanical removal and yielding superior surface finish (Ra < 1 nm) at a relatively lower MRR. Thus, abrasive selection involves a trade-off between removal rate and surface quality, depending on workpiece material and processing stage.

Studies have shown that increasing the abrasive concentration is beneficial for enhancing contact force and normal force in the initial stage. However, when the concentration exceeds the optimal range of 5–10%, it instead reduces the impact force, prolongs the system response time, and increases surface roughness due to uneven friction [54]. The effect of abrasive particle size on polishing performance also exhibits an optimal characteristic: with a fixed CIP particle size, smaller abrasives can achieve a smoother surface. However, when the abrasive particles become too small, the impact force becomes insufficient and energy is dissipated, leading to a decrease in MRR [25]. Moreover, the relative size relationship between abrasives and CIP particles is also crucial. Nagdeve et al. [54] found that when CIP and abrasive particles are comparable in size, the MR polishing fluid exhibits a higher yield stress, which is beneficial for improving material removal efficiency. The non-monotonic effects of polishing particle volume fraction and size on yield stress observed in the above experiments were theoretically explained by the microscopic constitutive model of Song et al. [55]. The model indicates that when the volume fraction of polishing particles is low, they can fill the inter-chain gaps, thereby reinforcing the chain structure and increasing the yield stress. However, an excessively high volume fraction causes excessive intrusion into the CIP chains, increasing the inter-chain spacing and drastically reducing the magnetic interaction force, which in turn decreases the yield stress. Similarly, there exists an optimal polishing particle radius (approximately 0.7–1 times the CIP radius): particles that are too small cannot effectively clamp the chains, while those that are too large lead to chain breakage. These theoretical predictions are consistent with the experimentally optimized results, providing quantitative guidance for the selection of process parameters.

(4) Additives include surfactants, dispersants, stabilizers, and chemical activators, among others. Although used in small quantities, they play a critical role in improving the overall performance of the polishing fluid. Surfactants (e.g., oleic acid, stearic acid, polyethylene glycol, sodium dodecylbenzenesulfonate (SDBS), etc.) can adsorb onto particle surfaces and inhibit agglomeration through steric hindrance effects. Dispersants (e.g., sodium hexametaphosphate) can improve the uniform dispersion of particles in the carrier liquid. Thixotropic agents (e.g., fumed silica) can adjust the viscosity and thixotropy of the system, thereby slowing down the sedimentation rate of magnetic particles [56,57,58].

#### 2.1.2. Nonlinear Rheological Models

The most distinctive feature of MR polishing fluid is its nonlinear rheological behavior under an external magnetic field. Under zero magnetic field conditions, MR polishing fluid can be regarded as a Newtonian fluid, with shear stress exhibiting a linear relationship with shear rate. When an external magnetic field is applied, the magnetic particles align along the magnetic field lines to form chains, transforming the fluid into a viscoplastic body with a yield stress, while the apparent viscosity shows nonlinear changes [59]. Accurately describing and predicting this nonlinear rheological behavior is of great significance for establishing material removal models, optimizing process parameters, and achieving precision control of the processing.

Currently, scholars have proposed various nonlinear models to characterize the rheological behavior of MR polishing fluids, among which the most commonly used include the Bingham plastic model, the Herschel–Bulkley model, the Casson model, and the biviscosity model, among others.

The Bingham plastic model is the most fundamental model for describing the rheological properties of MR polishing fluid [14]. This model assumes that the fluid begins to flow only when the applied shear stress exceeds the critical yield stress $\tau_{0}$; beyond this threshold, the shear stress exhibits a linear relationship with the shear rate:

(1) $$\tau \;={\;\eta }_{0}\dot{\gamma \;}+{\;\tau }_{0}\mathrm{sgn}(\dot{\gamma })$$

where τ is the shear stress, $\eta_{0}$ is the plastic viscosity, $\dot{\gamma }$ is the shear rate, and $\tau_{0}$ is the yield stress. The Bingham model is simple in form and involves few parameters, making it suitable for approximate calculations in the moderate-to-high shear rate range. However, its main drawback is the inability to describe the nonlinear characteristics of fluid behavior at low shear rates, as well as the singularity problem at the yield surface in numerical simulations. To overcome this limitation, the Bingham-Papanastasiou model eliminates the discontinuity in numerical calculations by introducing an exponential smoothing term [60].

The Herschel–Bulkley model is a generalization of the Bingham model, introducing power-law behavior to describe shear-thinning or shear-thickening phenomena after yielding [38]:

(2) $$\tau \;={\;\tau }_{0}\;+\;k{\dot{\gamma }}^{n}$$

where k is the consistency index and n is the flow behavior index. When n < 1, the fluid exhibits shear-thinning; when n > 1, it exhibits shear-thickening; and when n = 1, the model reduces to the Bingham model. The Herschel–Bulkley model has broader applicability and can more accurately describe the rheological behavior of MR polishing fluid over a wide range of shear rates, and it is considered the most physically meaningful comprehensive model.

The Casson model was originally developed to describe the rheological behavior of suspension systems (e.g., paints, blood) and was later introduced into the field of magnetorheological fluids [39]. The model takes the following form:

(3) $$\sqrt{\tau }\;=\;\sqrt{\tau_{y}}\;+\;\sqrt{\eta_{\infty }\dot{\gamma }}$$

where $\tau_{y}$ is the Casson yield stress, and $\eta_{\infty }$ is the infinite shear viscosity. The Casson model exhibits good predictive capability at very low shear rates, but its performance is relatively poor at high shear rates.

The biviscosity model [6] introduces two different viscosity coefficients (*η*_1_ and *η*_2_) to describe the rheological behavior before and after yielding, respectively, thereby enabling a more accurate characterization of the transition from elastic to plastic flow. However, this model involves a relatively large number of parameters, requiring extensive experimental data for calibration.

In practical applications, the choice of model depends on the processing conditions and accuracy requirements. Table 2 summarizes the characteristics of several models [38].

### 2.2. Key Performance Indicators and Their Optimization

#### 2.2.1. Sedimentation Stability

Sedimentation stability is one of the key indicators for evaluating the practical application value of MR polishing fluid. Due to the significant density difference between magnetic particles and the carrier liquid, magnetic particles gradually settle under gravity, leading to stratification or even hardening of the polishing fluid, which severely affects processing stability and the service life of the polishing fluid [61]. Therefore, how to improve the anti-sedimentation performance of MR polishing fluid is a research focus in this field.

The sedimentation process is primarily governed by interparticle interactions. During static storage, van der Waals attraction and magnetic dipole interactions between magnetic particles lead to particle agglomeration, forming larger aggregates that accelerate sedimentation. The core strategy to address this issue is to enhance steric hindrance or electrostatic repulsion between particles through surface modification or the addition of dispersants.

Surfactant modification is the most commonly used approach. By adsorbing long-chain organic molecules onto the surface of magnetic particles, a solvation layer is formed that effectively prevents direct contact between particles. Xiong et al. [56] prepared MR polishing fluids using three surfactants with different HLB values—oleic acid, polyethylene glycol, and sodium dodecylbenzenesulfonate (SDBS)—and evaluated the anti-sedimentation stability by measuring the influence of the dispersed particles on wettability. They found that the surfactant with a lower HLB value exhibited better anti-sedimentation performance. Shu et al. [57] modified hydroxyl iron powder with the nonionic surfactant polyethylene glycol (PEG); the resulting water-based MR polishing fluid achieved a minimum sedimentation rate of 8.56%, and its original performance could be restored after re-stirring. Polishing experiments on K9 glass using this fluid demonstrated a volume removal rate exceeding 1.4 mm^3^/min.

Nanoparticle addition is another effective strategy. By introducing nanoscale particles (such as nano-SiO_2_, Fe_3_O_4_, carbon nanotubes, etc.) into the magnetic particle suspension system, these nanoparticles fill the gaps between micron-sized magnetic particles, forming a three-dimensional network structure that provides support and buffering, thereby slowing down sedimentation. Piao et al. [58] synthesized Fe_3_O_4_/SiO_2_ core–shell nanoparticles using a sol–gel method and added them to the MR polishing fluid. They found that the sedimentation rate was lower than that of pure MR polishing fluid, and the rheological properties were also improved. Guo et al. [59] investigated the effects of nano-SiO_2_ with three different specific surface areas (150, 200, and 380 m^2^/g) on the performance of MR polishing fluid. They found that the addition of SiO_2_ induced gelation to form a stable three-dimensional network structure, which reduced the fluidity of the MR polishing fluid to a certain extent but significantly retarded particle sedimentation.

The composite surfactant system can produce synergistic effects. Yang et al. [62] prepared an MR polishing fluid using dimer acid as a surfactant and found that the dimer acid could form a loose flocculated structure, thereby enhancing sedimentation stability. Niu et al. [63] used oleic acid and polymethyl methacrylate (PMMA) as composite surfactants, respectively, and demonstrated that both can provide a strong steric hindrance effect to inhibit particle agglomeration.

#### 2.2.2. Shear Yield Stress and Magnetic Responsiveness

Shear yield stress is the minimum stress required for the MR polishing fluid to transition from a liquid to a solid state under a magnetic field. It is a core parameter for evaluating the strength of the magnetorheological effect and directly affects the material removal capability during polishing [10]. Under the same magnetic field conditions, a higher yield stress indicates a “harder” polishing fluid, which provides greater clamping force on the abrasives and transfers larger normal pressure and shear force to the workpiece surface, thereby achieving a higher material removal rate.

The volume fraction of magnetic particles has the most significant effect on the yield stress. Sun et al. [64] prepared four MR polishing fluids with volume fractions of 10%, 20%, 30%, and 40%, respectively, and measured their shear stresses at different shear rates using a rheometer. They found that under the same magnetic field and shear rate, a higher volume fraction led to a higher shear stress. This is because a higher solid content implies that more chain-like structures can form under the magnetic field, thereby enhancing the overall mechanical strength of the system.

The particle size of magnetic particles also has a significant effect on the yield stress. Theoretical and experimental studies by Ginder and Davis [40] have shown that in the small particle size range, the yield stress increases significantly with increasing particle size, which is related to the stronger magnetic dipole interaction forces between larger particles. However, when the particle size exceeds a certain critical value (approximately 10 μm), the magnetic domain structure of the particles themselves tends to stabilize, and the yield stress no longer changes markedly with further increases in particle size. Therefore, in engineering practice, carbonyl iron powder with a particle size in the range of 2–8 μm is typically selected as the magnetic particle component.

The external magnetic field strength is the most critical externally controllable parameter for adjusting the yield stress. Xiao et al. [65] systematically investigated the microstructural changes of a silicone oil-based MR polishing fluid under different magnetic field strengths (0–150 mT) and their corresponding shear mechanical properties. They found that at low currents (0–1 A), there exists an exponential relationship between the shear stress of the magnetorheological fluid and the applied current, with an exponent of approximately 1.5. When the current further increases, the shear stress transitions to a linear growth and eventually reaches a saturation value. This indicates that there is an optimal range of magnetic field strength, beyond which the marginal gain diminishes.

The type of carrier liquid also has an important influence on the magnetorheological effect. By fitting the Bingham plastic model, Li [66] compared the variation of shear yield strength with magnetic field for two polishing fluids: an ionic liquid-based (IL-MRF) and a silicone oil-based (SO-MRF) magnetorheological fluid. The results showed that under a relatively high applied magnetic field (436 kA/m), the yield strength of IL-MRF was approximately 29% higher than that of SO-MRF, exhibiting a stronger magnetorheological effect. This finding provides new insights for the development of novel high-performance carrier liquid systems for magnetorheological fluids.

### 2.3. Novel High-Performance Polishing Fluids

As the application fields of MRF technology continue to expand, the performance requirements for polishing fluids are becoming increasingly demanding. Aerospace and advanced optics require high-performance polishing fluids with ultra-high material removal capability and excellent stability. On the other hand, considering manufacturing cost requirements, it is necessary to develop polishing fluids that combine both stability and economy.

The development of core–shell structured composite magnetic particles represents an important direction for high-performance polishing fluids. Machovsky et al. [46] prepared CI/ZnO core–shell particles, which significantly enhanced the thermal-oxidative stability, sedimentation stability, and magnetorheological performance of the magnetorheological fluid, and for the first time demonstrated that a dual-morphology (pure CI+core–shell particles) hybrid system can achieve even higher yield stress through synergistic effects. Mrlik et al. [49] coated CIP with cholesteryl chloroformate, improving compatibility with silicone oil-based carrier liquids and thermal stability. CI/PMMA core–shell particles substantially reduce density (approximately 2.31 g/cm^3^), improve redispersibility, and significantly enhance sedimentation stability. In addition, composite systems such as CI/γ-Fe_2_O_3_, CI/PS (polystyrene), and CI/PANI/MWCNT (polyaniline/multiwalled carbon nanotubes) have all exhibited excellent comprehensive performance [67].

Chemically enhanced polishing fluids promote chemical reactions during polishing by adding chemically active components to conventional formulations. For example, Deng et al. [68] developed a highly catalytically active water-based magnetorheological chemical finishing fluid (HCAMRCFF) using Fe_3_O_4_ and Cr as composite catalyst particles, oleic acid and polyethylene glycol as surfactants, and H_2_O_2_ as an oxidizing agent for polishing single-crystal SiC. Compared with the unmodified polishing fluid, the catalytic activity was improved by 65.4%, the MRR was increased by 72.5%, and the surface roughness was reduced by 13.22%. Zhang et al. [69] formulated a polishing fluid in a triethanolamine (TEA) alkaline system that can chemically react with fused silica surfaces, achieving a high material removal rate of 10.0 λ/min, while utilizing the steric hindrance effect of ethanol ligands to obtain an ultra-smooth surface with a root mean square (RMS) roughness of 0.46 nm.

In summary, the development of MR polishing fluid has evolved from merely pursuing a high removal rate to a multi-objective optimization process that comprehensively considers processing performance, stability, and economy. In the future, such novel intelligent polishing fluids will become an important research direction in this field.

## 3. Mechanism and Theoretical Models of Magnetorheological Finishing

### 3.1. Material Removal Mechanism

The material removal in the MRF process is the result of the synergistic effects of mechanical action, chemical action, and rheological effects, rather than a simple superposition of a single mechanism [70]. An in-depth understanding of the material removal mechanism is of great significance for establishing accurate theoretical models, optimizing process parameters, and improving processing efficiency and quality.

#### 3.1.1. Macroscopic Mechanical Mechanism

During the MRF process, from a macroscopic perspective, the material removal mechanism can be attributed to three fundamental actions: mechanical scratching action, chemical corrosion action, and rheological extrusion action [71].

The mechanical scratching action is the most direct method of material removal. When the MR fluid is delivered to the rotating wheel and enters the converging gap between the wheel and the workpiece, the magnetic field causes the fluid to form a “hardened zone.” The normal magnetic force (penetration force) is transmitted through the chain-like structure to the abrasive particles, pressing them into the workpiece surface. Subsequently, due to the relative motion between the wheel and the workpiece, shear action occurs between the abrasives and the workpiece surface, and material is removed in the form of micro/nanoscale chips, thereby achieving nanoscale surface finish [72]. This mechanism is primarily governed by surface shear stress rather than the normal indentation dominance in conventional polishing. The energy provided by the shear stress facilitates the formation of chemical bonds between the active centers of polishing crystals and the atoms (e.g., silicon atoms) on the workpiece surface, thus removing material through adsorption–desorption, which can avoid subsurface damage.

The intensity of the mechanical scratching action mainly depends on the shear stress τ and normal pressure P at the interface between the polishing film and the workpiece. Kordonski and Jacobs [14] first proposed that material removal in MRF mainly originates from the shear stress generated by shear flow, rather than from pure indentation. Subsequent studies have also shown that the MRF polishing film exhibits significant non-Newtonian flow characteristics, and its shear stress distribution is closely related to multiple factors such as the applied magnetic field strength, the working gap, and the fluid viscosity.

The chemical corrosion action refers to the reaction between active components in the polishing fluid and the workpiece surface material, forming a soft corrosion layer with lower hardness that is easily removed mechanically [73]. Taking quartz glass as an example, in an alkaline MR polishing fluid, SiO_2_ on the glass surface reacts with OH^−^ as follows:

(4) $$\mathrm{Si}O_{2}\;+\;2OH^{-}\;\to \;\mathrm{Si}{O^{2-}}_{3}\;+\;2H_{2}O$$

The generated silicate ions (SiO_3_^2−^) continue to hydrolyze to form silicic acid, part of which polymerizes into polysilicic acid, while the other part forms silicic acid colloid. The hardness of this corrosion layer covering the workpiece surface is much lower than that of the bulk material. As polishing proceeds, the layer is continuously removed by abrasives, exposing fresh surfaces that are further corroded [71]. This “corrosion–removal–recorrosion” cyclic process greatly improves the material removal efficiency.

The key to promoting the chemical action lies in the design of the polishing fluid formulation, including the selection of suitable pH buffers, oxidizers (e.g., H_2_O_2_), and catalysts (e.g., Fe^2+^) [74].

The rheological extrusion action refers to the plastic extrusion and “leveling effect” on the micro-asperities of the workpiece surface caused by the normal pressure exerted by the polishing film during flow [73]. This mechanism results in almost no substantial material removal; instead, it reduces the microscopic roughness by inducing micro-zone plastic flow and redistribution of the surface material. The rheological extrusion action has a significant influence on improving the surface finish of highly ductile materials, whereas its effect on high-hardness brittle materials is relatively limited.

In actual processing, these three mechanisms often coexist, but their relative importance depends on the properties of the workpiece material, the chemical activity of the polishing fluid, and the process parameters. For high-hardness, high-brittleness materials, mechanical scratching and chemical corrosion together play dominant roles; for ductile materials such as copper, all three mechanisms may act simultaneously [75].

#### 3.1.2. Atomic-Scale Material Removal Mechanism

As MRF technology advances toward sub-nanometer or even atomic-level surface accuracy, macroscopic continuum models have become insufficient for accurately describing material removal behavior within just a few atomic layers of the processed surface. Molecular dynamics (MD) simulations can play a key role in addressing this issue, revealing the microscopic interaction mechanisms at the abrasive–workpiece interface from the atomic/molecular level [76].

MD simulation of single abrasive grain scratching process: Wang et al. [77] established a molecular dynamics model of a single spherical abrasive (carbon) scratching the surface of a GaAs crystal in planar MRF, systematically investigating the evolution of surface morphology and subsurface damage under different indentation depths. The simulation results show that at an indentation depth of 1.5 nm, due to the elastic recovery of the GaAs substrate, no visible scratch appears on the surface, but local irreversible atomic configuration changes (i.e., plastic deformation) have already occurred in the subsurface region. As the indentation depth increases, a large number of atoms accumulate in front of and on both sides of the abrasive, forming ridges; the ridge height increases with the scratching distance. Simultaneously, the range of atomic bond breakage and structural changes expands deeper into the material, inducing atomic rearrangement, compaction by extrusion, and enlargement of the plastic deformation zone. These changes in the subsurface microstructure (e.g., an increase in the number of CD-1 and HD-1 type atoms) indicate continuous plastic deformation and evolution of the atomic structure within the material.

Abrasive geometry and size effect: Li et al. [78] systematically investigated the atomic accumulation behavior of spherical aggregated nanodiamond abrasives of different sizes (90 nm, 200 nm, 500 nm) under the same normal load ratio using molecular dynamics simulations. The results indicate that as the aggregate size increases (with a corresponding increase in the normal load), the height of atomic accumulation in front of and on both sides of the abrasive significantly increases, and the number of removed atoms gradually evolves from a monolayer to multi-atomic clusters. When the aggregate size increases from 90 nm to 500 nm, the contact area substantially increases, the indentation depth deepens, leading to wider and deeper scratch grooves, an enlarged atomic accumulation zone, and deteriorated surface roughness. Furthermore, during sliding, the larger aggregated particles cause the adsorbed atoms to form chain-like structures, whereas the smaller particles only achieve monolayer removal. This suggests that the abrasive aggregate size directly influences the material removal mechanism and surface integrity.

These atomic-scale simulation studies reveal the essence of material removal during the MRF process: (1) local high pressure and shear at the abrasive–workpiece interface induce atomic displacement and migration; (2) chemical reactions significantly lower the threshold stress for mechanical removal by weakening the interatomic bonding strength on the surface; and (3) by appropriately matching chemical activity and mechanical action intensity, an atomically smooth surface can be obtained without causing plastic damage. These findings provide an important theoretical basis for the development of chemical–mechanical magnetorheological composite finishing (CMMRF) technology.

### 3.2. Material Removal Function and Polishing Force Model

The fundamental reason why MRF technology is known as a deterministic machining method is that its material removal process can be quantitatively described and predicted through mathematical models. The material removal function, also referred to as the tool influence function (TIF), characterizes the distribution of material removal amount at each point on the workpiece surface per unit time by the polishing tool, and serves as the basis for dwell time calculation and form error correction [79]. The polishing force model establishes a quantitative relationship among process parameters, magnetic field distribution, and polishing pressure/shear force, and is a key prerequisite for forming an accurate removal function.

#### 3.2.1. Classical and Modified Preston Equation

The Preston equation is the most classical empirical formula for describing the material removal rate in optical polishing, and its basic form is

(5) MRR =KPVt

where MRR is the material removal amount, K is the Preston coefficient (which comprehensively reflects factors such as abrasive type, polishing fluid composition, and temperature), P is the polishing pressure, V is the relative velocity, and t is the polishing time.

In the early stage of MRF technology development, the Preston equation was generally used directly to describe the material removal behavior, with the normal pressure P considered the dominant factor affecting the removal rate [29]. As research progressed, researchers gradually recognized that shear stress plays a more critical role in the material removal mechanism of MRF.

Shear stress-based modified model: Miao et al. [18] modified the comprehensive coefficient K in the Preston equation and proposed to introduce the shear stress τ into the model, modifying the original equation as follows:

(6) $$\mathrm{MRR}\;=\;C_{P,\mathrm{MRF}(\tau )\;}\times \;\tau \;\times \;V$$

This modification emphasizes the direct correlation between MRF material removal and fluid shear stress.

Pressure-shear force synergistic model: Liu et al. [27] further proposed a removal rate model incorporating the synergistic effect of pressure and shear force:

(7) $$\mathrm{MRR}\;={\;C}_{P,\tau }P^{\alpha }(D,\eta ,U)\tau (D,\eta ,U)$$

This model expresses both the pressure P and shear stress τ in the polishing zone as functions of the working gap D, polishing fluid viscosity η, and polishing wheel linear velocity U, thereby more comprehensively reflecting the influence of process parameters on the removal rate. Experimental validation shows that the predictions of this model for material removal at different positions in aspheric surface machining are in good agreement with the measured results.

#### 3.2.2. Influence Function Modeling

Influence Function (TIF) is a quantitative characterization that describes the distribution of material removal by the polishing tool at various points in space per unit time, and serves as the core input parameter for achieving deterministic form correction. The accuracy of the influence function directly affects the precision of dwell time calculation and the convergence effectiveness of the final form error.

Influence Function Modeling of Wheel-Type Tools: Schinhaerl et al. [80] established a three-dimensional numerical model of the influence function for a wheel-type MRF tool based on the Preston law and a simplified pressure calculation method. By comparing the calculated influence function with the actual measured influence function from processing, a high degree of consistency was demonstrated, validating the effectiveness of this method. Dai et al. [81] introduced an efficiency coefficient to calibrate the model based on the above work, thereby improving the model’s adaptability and prediction accuracy under different processing conditions.

Yang et al. [82] proposed an influence function prediction method based on the spot breeding method. This method establishes a transformation mechanism between the immersion depth of the MR polishing fluid and the shape of the contact spot, enabling accurate prediction of the influence function shape under different immersion depths with relatively few calibration experiments, thereby greatly simplifying the calibration process of the influence function.

Regionalized Modeling for Freeform Surface Machining: Liu et al. [83] proposed an interactive regionalized influence function modeling method to address the specific issue in aspheric and freeform surface machining, namely that the traditional influence function is only applicable to flat or spherical workpieces and cannot predict the variation in material removal at different positions on an aspheric surface. This method divides the aspheric workpiece into multiple local zones and individually corrects the removal function for each zone based on the actual curvature and normal vector of that zone, thereby achieving accurate prediction of material removal distribution on complex curved workpieces.

Influence Function under Multi-Pole Dynamic Magnetic Field: In disk-type cluster MRF processing, Nie et al. [84] investigated the magnetic field distribution characteristics under various magnetic pole arrangements (same-direction arrangement, cross-layer arrangement, cross-line arrangement, and cross-orientation arrangement) and their influence on the shape of the influence function. By introducing magnetic field characteristic parameters based on magnetic flux density—namely $B_{\mathrm{rms}}$ (overall strength), $B_{\mathrm{avg}}$ (average magnetic flux density), $B_{\mathrm{flat}}$ (uniformity), and $B_{\mathrm{fluc}}$ (dynamic characteristics)—the polishing uniformity of each arrangement scheme was quantitatively evaluated:

(8) $$B_{\mathrm{rms}}=\sqrt{\frac{1}{R}\int_{0}^{R}{\left(\frac{1}{2\pi r}\oint B_{m}\left(r,\theta \right)\;d\theta \right)}^{2}\mathrm{dr}}$$

(9) $$B_{\mathrm{avg}}=\frac{1}{\pi R^{2}}\int_{0}^{R}\oint B_{m}(r,\theta )\;d\theta \mathrm{dr}$$

(10) $$B_{\mathrm{flat}}=\sqrt{\frac{1}{R}\int_{0}^{R}{\left(\frac{1}{2\pi r}\oint B_{m}\left(r,\theta \right)\;d\theta -B_{\mathrm{avg}}\right)}^{2}\mathrm{dr}}$$

(11) $$B_{\mathrm{fluc}}=\sqrt{\frac{1}{2\pi }\oint {\left(\frac{1}{R}\int_{0}^{R}B_{m}\left(r,\theta \right)\;\mathrm{dr}-B_{\mathrm{avg}}\right)}^{2}\;d\theta }$$

where r represents the radial distance in polar coordinates, θ represents the azimuth angle in polar coordinates, and R denotes the radius of the magnetic field action region. Both simulation and experimental results indicate that the cross-orientation arrangement is superior to the other arrangements in terms of magnetic flux density magnitude, flatness, and fluctuation, which is conducive to achieving a uniform material removal distribution.

In summary, research on influence function modeling exhibits several trends: (1) evolution from two-dimensional models to three-dimensional models; (2) development from pressure-based empirical models to multi-physics field hybrid models that incorporate fluid mechanics and magnetics; and (3) progression from a unified influence function for simple surface shapes to regionalized influence functions for complex curved surfaces.

#### 3.2.3. Prediction Accuracy and Error Analysis of the Removal Function Model

The accuracy of the material removal function model directly determines the calculation precision of dwell time and the convergence effect of deterministic processing. In recent years, researchers have begun to systematically evaluate the prediction errors of removal function models and develop higher-precision modeling methods.

In the area of removal function models based on shear stress prediction, Tien et al. [85] combined an improved Halbach array with a slider-crank mechanism to establish a magnetorheological polishing force model for Ti-6Al-4V alloy. They also developed a material removal rate prediction model based on Preston’s equation, finding that models considering only normal force or only tangential force had insufficient prediction accuracy, whereas a modified model incorporating both forces showed good agreement with experimental results [86]. Liu et al. [85] integrated rheological theory and granular flow theory to establish a layered stress model, decomposing the normal pressure in the polishing zone into a compressive stress driven by yield stress and a collisional stress controlled by the Bagnold number. They introduced the Boltzmann distribution to describe the spatial distribution of abrasive particle concentration, and the resulting removal function model demonstrated high prediction accuracy in planar component polishing experiments [85].

In terms of model prediction errors, Tian et al. [87] developed an elasticity-enhanced magnetorheological shear-thickening polishing (MRSTP) material removal rate model, achieving an average prediction error of less than 5.0% between theoretical values and experimental results, with a maximum removal rate of 3.3 μm/h. Wang et al. [88] achieved a model prediction deviation of only 2.31% for material removal rate and 5.12% for surface roughness through response surface methodology optimization. In magnetorheological foam plane polishing (MRFPF), the MRR model established based on workpiece trajectory updating method, magnetic field distribution, and polishing speed yielded a relative error between theoretical and experimental results of less than 11.63% [89]. These results indicate that current removal function models already achieve good engineering prediction accuracy for planar or nearly planar workpieces, but their predictive capability for complex curved surfaces still needs improvement.

## 4. Magnetorheological Polishing Tools and Hybrid Machining Technology

### 4.1. Typical MRF Tools and Systems

With the continuous maturation of MRF technology and the expansion of its application fields, researchers have developed various structural configurations of MRF tools to meet the processing requirements of workpieces with different shapes, sizes, and materials. Based on the geometric morphology of the polishing tool and its contact mode with the workpiece, typical MRF tools can be classified into wheel-type, ball-end-type, disk-type/cluster-type, and other forms for special structural processing [6,7,10,34]. Table 3 summarizes the types of workpieces suitable for each category of tool.

#### 4.1.1. Wheel-Type Magnetorheological Finishing

**Figure 3:**
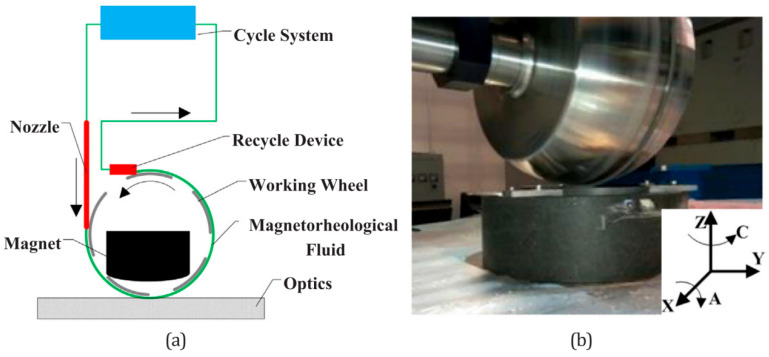
MRF device. (**a**) Component nomenclature and arrangement. (**b**) MRF working wheel [90].

Wheel-type MRF is the most mature, commercially available, and widely used form of MRF [90]. Its working principle (Figure 3) is as follows: an electromagnet or permanent magnet is placed inside the polishing wheel. When energized, it generates a high-gradient magnetic field in the gap between the polishing wheel and the workpiece. The circulation system delivers the MR polishing fluid to the surface of the polishing wheel. As the polishing fluid passes through the magnetic field, it undergoes a rheological effect, forming a layer of viscoplastic flexible polishing film (polishing ribbon) on the wheel surface. The rotation of the polishing wheel drives the polishing film to produce relative motion against the workpiece surface, and the shear stress at the interface achieves microscale material removal.

In terms of equipment development, the smallest polishing wheel developed by QED Technologies has a diameter of only 10 mm and can process tiny and complex components; the largest polishing wheel has a diameter of 370 mm and can process concave lenses up to 2.3 m and convex lenses up to 1.7 m [91]. These systems are all integrated with a CCOS (Computer Controlled Optical Surfacing) computer control system, enabling deterministic corrective machining of the workpiece surface form.

The Peng team [92] designed an inverted four-axis linkage wheel-type polishing equipment, successfully converging the PV value of optical glass components from 261.7 nm to 55.3 nm and the RMS value from 46.8 nm to 5.5 nm. Li et al. [90] integrated the polishing unit into a five-axis CNC machining center, achieving high-precision processing of large-aperture aspherical optical elements. On the commercialization front, domestic wheel-type MRF equipment such as the MRP series, DQ series, and ZJY-NCM series have been introduced to the market, marking the entry of this technology into the practical application stage.

In terms of process research, focusing on core issues such as material removal efficiency, processing stability, and form correction accuracy, Byung et al. [93] systematically analyzed the effects of magnetic induction intensity, carbonyl iron powder volume fraction, polishing depth, and other factors on the removal rate through a five-factor, two-level orthogonal experiment. The experiments revealed that magnetic field intensity has the most significant effect on the removal rate, followed by polishing depth and polishing time, while the effect of CIP concentration on the removal rate is not significant. Li [24] utilized a self-developed MRF360 wheel-type polishing equipment to verify the feasibility of efficient deterministic machining of large-aperture aspheric surfaces using MRF.

In terms of theoretical modeling, Bai et al. [94] established a calculation method for the normal pressure and effective friction force in the polishing zone based on continuum medium theory and dense granular flow theory, and on this basis proposed a new material removal model for MRF. This model effectively decouples the main process parameters affecting material removal and reveals the generation mechanism of shear force on the polished workpiece. The interactive regionalized modeling method proposed by Liu et al. [83] successfully solved the spatial adaptability problem of the influence function on aspheric surfaces.

Wheel-type MRF offers the greatest advantages in its integration with CCOS and deterministic processing capability, enabling ultra-precision finishing of optical components such as large-size, aspheric, and complex curved surfaces. However, it also has certain limitations: (1) The contact between the polishing wheel and the workpiece is point contact, so the number of abrasive particles involved in material removal per unit time is limited, resulting in a relatively low material removal rate. (2) The motion trajectory of the polishing wheel is a single path, and overlap errors between adjacent trajectories can easily occur, causing periodic replication of form errors. (3) The equipment requires high precision, typically necessitating large, high-precision machine tools, which are expensive. (4) Constrained by the geometric size of the polishing wheel, currently it can only process aspheric components with an aperture larger than 8 mm [95].

#### 4.1.2. Ball-End Magnetorheological Finishing

**Figure 4:**
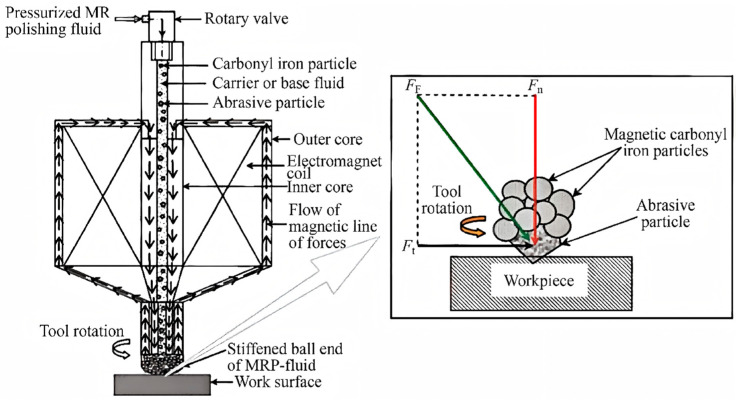
Principle of ball-type magnetorheological polishing technology [10].

The ball-end type MRF employs a spherical polishing tool with a relatively small diameter to achieve material removal through point contact, making it particularly suitable for processing small optical components with large radii of curvature and complex curved surfaces [96]. Its working principle (Figure 4) is as follows: the MR polishing fluid enters the processing zone from above the polishing head through an enclosed magnetic tube, and after being magnetized in the gradient magnetic field generated by a permanent magnet or electromagnet, it forms a spherical solid-like flexible polishing film on the surface of the polishing ball head. Driven by the rotating ball head, the polishing film undergoes shear motion against the workpiece surface, thereby achieving micro material removal.

In terms of equipment development, Singh et al. [97] developed a ball-end type MRF device based on electromagnet excitation, where the normal pressure of the polishing film against the workpiece surface is controlled by adjusting the excitation current of the coil. Sidpara and Jain [98] replaced the electromagnet with a permanent magnet, making the device more convenient and practical. Using a three-axis CNC milling machine as the driving unit, they performed magnetorheological finishing on a complex freeform surface knee joint implant. They measured normal force, tangential force, and axial force in real time, investigated the effects of curvature angle, tool rotation speed, and feed rate on these forces, and established theoretical models for the normal and tangential forces.

**Figure 5:**
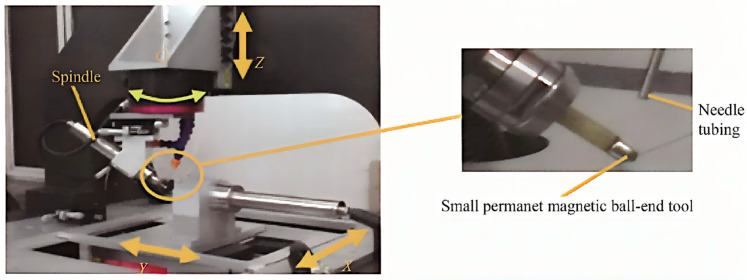
Small-diameter permanent magnet ball head magnetorheological polishing prototype [10].

The Chen team [99] developed a small-diameter permanent magnet ball-end MRF prototype (Figure 5), using a permanent magnet as the excitation source and adopting four-axis linkage control. This design makes the polishing head more flexible in posture, enabling it to adapt to the processing of workpieces with various curvatures.

**Figure 6:**
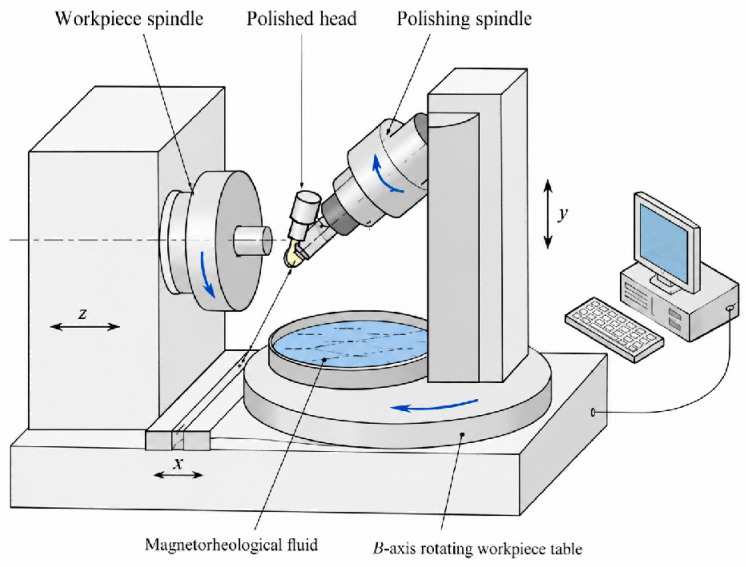
Slant axis magnetorheological polishing system [100].

Yin et al. [100] further developed a tilted-axis ball-end MRF equipment (Figure 6). By adjusting the tilt angle between the polishing head and the normal direction of the workpiece, this design effectively avoids structural interference issues during processing.

In addition, Wang et al. [101] introduced ultrasonic vibration assistance based on the ball-end equipment and developed an ultrasonic–magnetorheological hybrid polishing device. Ultrasonic vibration effectively improves the material removal efficiency and the processed surface quality by increasing the contact frequency between the MR polishing fluid and the workpiece surface, as well as the complexity of the abrasive particle motion paths.

**Figure 7:**
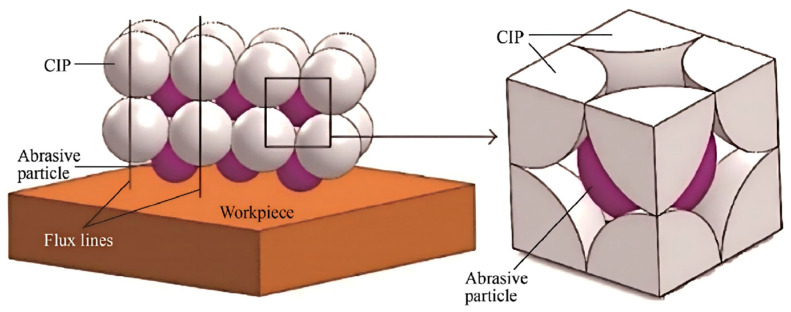
Exaggerated view microstructure arrangement and a unit cell with a BCC structure [10].

Theoretical and Process Research: Zafar and Jha [102] proposed an abrasive-CIP spatial distribution model based on a body-centered cubic (BCC) lattice structure (Figure 7), and on this basis established a theoretical prediction model for the surface roughness in ball-end MRF processing. Chen et al. [28] developed a three-dimensional hydrodynamic model of the polishing zone for a small-diameter permanent magnet ball-end MRF setup, concluding that material removal is dominated by shear stress. Tian et al. [103] investigated the effect of MR polishing fluid temperature on the material removal rate and found that increasing the temperature within the range of 20–60 °C reduces the fluid viscosity and improves its flowability, thereby significantly enhancing both polishing efficiency and processing quality.

The advantage of ball-end MRF lies in its strong processing flexibility, as it is not restricted by the shape or size of the workpiece, making it particularly suitable for finishing small-aperture optical components with irregular surfaces. The limitation is that the point-contact mode results in a relatively low material removal rate, rendering it unsuitable for efficient mass production. Moreover, for processing workpieces with complex curved surfaces, a more precise displacement control system is required to ensure accurate positioning of the polishing head, which increases both processing difficulty and cost.

#### 4.1.3. Disk-Type/Cluster-Type Magnetorheological Finishing

Disk-type/Cluster-type MRF adopts an “area contact” processing mode, which improves material removal efficiency by enlarging the contact area between the polishing film and the workpiece surface. This method is mainly applied to the ultra-precision planarization of large planar optical components [104].

**Figure 8:**
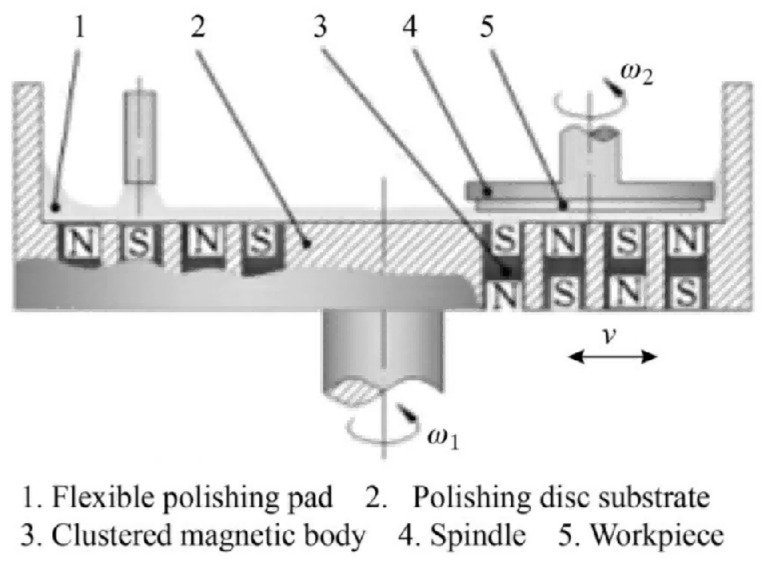
Principle of cluster-type magnetorheological polishing [33].

Working principle and equipment development: The Yan team [33] proposed the concept of cluster MRF (Figure 8). The principle involves arranging multiple permanent magnets uniformly beneath the polishing disk according to the cluster principle, thereby forming a high-intensity gradient magnetic field region containing multiple micro-abrasive heads. As the workpiece rotates with the spindle, shear motion occurs between the workpiece surface and the micro-abrasive heads, enabling high-efficiency, high-flatness ultra-smooth finishing.

The development of cluster MRF technology can be divided into four stages: (1) Static magnetic field stage: Fixed permanent magnet arrangements are used, and the magnetorheological polishing pad lacks self-repairing ability, resulting in limited processing efficiency and uniformity. (2) Dynamic magnetic field stage: Pan et al. [26] and Guo et al. [105] introduced a dynamic magnetic field by synchronously eccentrically rotating the magnetic poles, overcoming the drawbacks of difficult pad recovery and abrasive accumulation under a static magnetic field. (3) Variable-gap dynamic pressure stage: Yan et al. [106] proposed a variable-gap dynamic pressure cluster MRF method, in which an additional axial squeezing vibration of the workpiece during processing forces the magnetic chains to form a more robust body-centered tetragonal (BCT) configuration. This generates greater polishing pressure and abrasive-binding force, increasing the MRR by 19.5% and reducing the surface roughness by 42.96%. (4) Circular-hole array polishing plate stage: Luo et al. [107,108] machined a circular-hole array on the polishing plate, making the polishing shear force more uniform and stable.

In addition, the Yin team [12] developed permanent magnet/electromagnetic disk-type MRF equipment based on a large polishing mode, adopting an “upper workpiece, lower polishing disk” configuration where the workpiece is fixed on the upper spindle and the polishing disk rotates below, with the magnetic field provided by permanent magnets or DC electromagnets. Wu et al. [109] proposed a method of generating a dynamic magnetic field using low-frequency AC electromagnet excitation, which effectively improved the processed surface quality. Hu et al. [110] introduced an MR polishing fluid circulation system for the first time in disk-type equipment, which is of great significance for ensuring stable polishing fluid performance and processing consistency.

**Figure 9:**
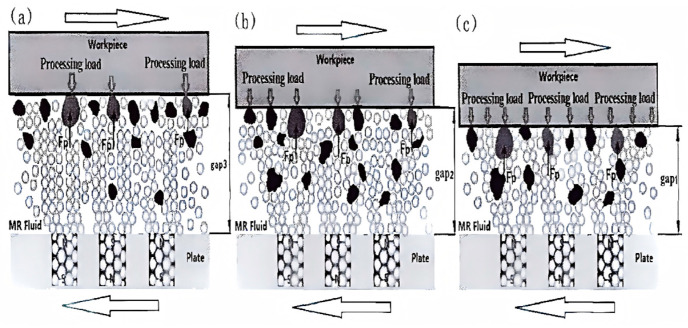
Schematic of accommodate-sinking process: (**a**) Contact (**b**) Embedding (**c**) Submergence [111].

Theoretical and Process Research: Pan et al. [26] established a material removal rate model for cluster MRF based on fluid mechanics principles and the Preston equation. Luo et al. [104] developed theoretical models for the shear force of solid particles and the material removal rate in MPF, analyzing the influence of circular-hole array parameters on polishing performance. Bai et al. [111,112] discovered an “embedding” effect of abrasive particles in cluster MRF (Figure 9): within a micro-abrasive head, only the top portions of abrasive particles of different sizes come into contact with the workpiece surface, while smaller particles automatically “fill in” the gaps left by larger particles, effectively enhancing the ultra-smooth planarization effect of the processed surface.

The advantage of disk-type/cluster MRF lies in its “area contact” mode, which increases the effective processing area and significantly improves the material removal efficiency. The dynamic magnetic field enhances the self-repairing ability of the polishing pad and the processing uniformity. The process theory is relatively well-established and mature. The limitation is that it is only suitable for processing planar or near-planar workpieces, and is difficult to adapt to curved surfaces, especially freeform surfaces. Moreover, it is difficult to achieve continuous circulation and renewal of the polishing fluid, which affects processing stability and accuracy.

#### 4.1.4. Other Forms of MRF Tools

In addition to the three mainstream configurations described above, researchers have developed various other forms of MRF tools to meet specific processing requirements (Table 4).

Rotary MRF tools are mainly used for polishing the inner walls and bottom surfaces of blind-hole cavity components [113]. A cylindrical electromagnet is fixed to the spindle, and the MR polishing fluid is distributed around the electromagnet to form a polishing body. The rotation and up-and-down motion of the polishing body simultaneously remove material from the inner wall and the bottom surface of the blind hole. Aggarwal and Singh [114] applied this technology to machine cylindrical blind holes, achieving a reduction in surface roughness of approximately 62.5% on the inner wall and 73.9% on the bottom surface, respectively. Yahya et al. [115] further introduced ultrasonic vibration assistance and verified the significant improvement in processing efficiency for the inner walls of aluminum alloy tubes using this technique.

Belt-type MRF tools use a closed magnetic pole box as the excitation source. By increasing the radius of curvature of the polishing zone, the effective contact area is enlarged, and this method is mainly used for high-efficiency processing of large-diameter planar optical elements [116]. Process experiments by Ren et al. [116] show that the material removal rate (MRR) for BK7 glass reaches as high as 2.52 μm/min, and for SiC reaches 1.22 μm/min, which is more than five times higher than that of conventional wheel-type equipment. However, this technique suffers from a single polishing direction, and obvious polishing marks tend to remain on the processed surface, which limits further improvement of surface quality.

Reciprocating MRF tools eliminate processing marks and improve surface quality through the reciprocating motion of the magnetic field generating device [117]. Wang et al. [89] replaced the permanent magnet with an electromagnet, which increased the material removal rate by 67% (reaching 44.3 nm/min). They also established prediction models for MRR and Ra using response surface methodology, with model prediction deviations of only 2.31% and 5.12%, respectively. This technology features a simple equipment structure and low cost, but the overall material removal rate is relatively low, and the machinable materials are currently limited to optical glass.

### 4.2. Advanced Hybrid MRF Technology

In the face of increasingly diverse material systems and ever-increasing precision requirements, conventional single-energy-field MRF processing has gradually revealed certain inherent limitations (Table 5). To address these issues, researchers have introduced auxiliary energy fields such as ultrasound, electrochemistry, laser, and chemistry, and have developed various multi-energy-field hybrid MRF technologies [118,119,120,121], opening up new directions for the advancement of MRF technology.

#### 4.2.1. Ultrasonic Vibration-Assisted MRF (UA-MRF)

Ultrasonic vibration-assisted MRF applies high-frequency (typically 20–40 kHz) and low-amplitude (from a few micrometers to tens of micrometers) ultrasonic vibrations to the workpiece or the polishing tool on the basis of a conventional MRF processing system. The vibrational energy is used to enhance the microscopic interaction between the abrasive particles and the workpiece surface.

Working Principle and Equipment: Zhang et al. [30,122] have conducted extensive pioneering research and designed an ultrasonic magnetorheological hybrid finishing (UMC finishing) device suitable for planar/convex and concave surfaces. In UMC finishing, ultrasonic vibration is applied to the polishing head, generating high-frequency oscillation in the vertical direction (frequency 20 kHz, amplitude 5 μm), while a magnetic field acting on the polishing head causes the magnetorheological fluid to form a flexible polishing tool in the polishing zone. The workpiece can remain stationary or rotate. The high-frequency oscillation significantly increases the contact frequency and impact velocity between the abrasive particles and the workpiece surface, and complicates the motion trajectories of the abrasives, thereby improving both the material removal efficiency and the surface quality (post-polish surface roughness Ra can reach 4.0 nm or 2.892 nm). Zhai et al. [123] further developed a UA-MRF process using Fe_3_O_4_/SiO_2_ core–shell structured abrasives for finishing sapphire substrates, achieving excellent ultra-smooth surfaces.

**Figure 10:**
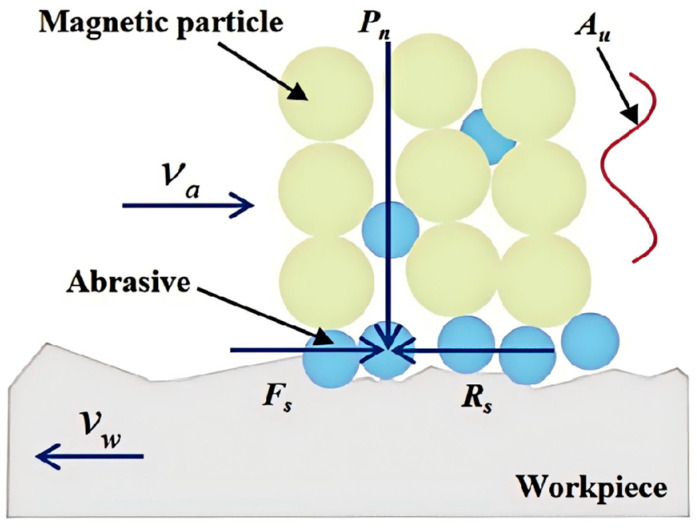
Schematic diagram of polishing process mechanism of UA-MRF [125].

Processing Characteristics and Mechanism (Figure 10): Through theoretical modeling and experimental validation, researchers have revealed the enhancement mechanisms of ultrasonic vibration on material removal: (1) Increased impact kinetic energy: Ultrasonic vibration imposes high-frequency (20 kHz) vertical oscillation on the workpiece or the polishing head. Under the combined action of the magnetic field and the ultrasonic field, the abrasive particles acquire high-acceleration impacts, increasing the instantaneous indentation depth and material removal. (2) Improved abrasive dispersion and fluid flow: Ultrasonic vibration promotes micro-turbulence and micro-jets in the magnetorheological polishing (MRP) fluid within the polishing zone, effectively reducing abrasive agglomeration, improving abrasive renewal frequency and distribution uniformity, and allowing more fresh abrasives to participate in cutting. (3) Thermo-chemical synergistic effect: The ultrasonic cavitation effect generates a large number of microbubbles in the polishing fluid, which collapse instantly, releasing high temperatures and shock waves that significantly accelerate the chemical reaction between the workpiece surface and the polishing fluid. Meanwhile, the local temperature rise reduces the viscosity of the MRP fluid, enhancing chemical activity and material removal efficiency. Experimental results show that under optimized conditions (ultrasonic frequency 20 kHz, amplitude 10–50 μm), the surface roughness Ra of sapphire is reduced from an initial 66 nm to 0.44 nm, and the material removal rate is increased by approximately 3.4 times compared with conventional MRF; the surface roughness of a titanium alloy nut is reduced from 1.247 μm to 0.104 μm; and the surface roughness Sa of an aluminum alloy cylindrical surface is reduced from 231.8 nm to 15.5 nm, with a material removal depth rate of 0.53 μm/min [124,125,126,127].

Mayank and Pulak [128] analyzed the contributions of ultrasonic power, polishing rotational speed, and abrasive concentration to the material removal rate in UA-MRF processing of single-crystal silicon through response surface experiments. They found that the contribution rate of ultrasonic power was as high as 57.9%, far exceeding those of polishing speed (13.3%) and abrasive concentration (12.5%), indicating the dominant role of ultrasonic vibration in this hybrid processing.

The advantage of UA-MRF lies in its significant improvement in material removal efficiency and surface quality, making it particularly suitable for difficult-to-machine materials with high hardness such as SiC and sapphire. The main limitation is that it is difficult to apply to large-volume and heavy-weight polishing tools, and it is currently mainly suitable for processing small- to medium-sized workpieces.

#### 4.2.2. Electro/Chemical-Assisted MRF (EC-MRF/CM-MRF)

Electro/chemical-assisted MRF modifies the physicochemical properties of the workpiece surface by introducing electrochemical or chemical reactions, thereby reducing the hardness and the difficulty of material removal, and consequently achieving high-efficiency and high-precision machining [71].

Electrochemical-assisted MRF (EC-MRF) removes material by applying an external voltage to induce anodic dissolution of the workpiece surface in an alkaline electrolyte, forming a loose oxide layer, which is subsequently removed by mechanical scratching. The advantage of this method lies in overcoming the difficulty of controlling chemical reactions in purely chemical MRF. Yan et al. [120] employed EC-MRF technology for ultra-smooth planarization of GaN wafers. After 3 h of processing, they achieved a damage-free surface with a surface roughness Ra of 0.6 nm, demonstrating significant potential for the precision machining of wide-bandgap semiconductor materials.

Chemo-mechanical-assisted MRF (CM-MRF) adds chemically active components such as H_2_O_2_, KOH, and Fenton‘s reagent (Fe^2+^+H_2_O_2_) to the MR polishing fluid to generate a chemical corrosion layer in situ on the workpiece surface. Liang et al. [74] introduced the Fenton reaction into magnetorheological finishing and established a chemo-mechanical hybrid MRF method for single-crystal SiC. The mechanism is as follows: Fe^2+^ catalyzes the decomposition of H_2_O_2_ to produce strongly oxidizing **·**OH radicals, which oxidize the SiC surface into a softened SiO_2_ layer; this layer is subsequently removed by the mechanical action of the abrasives, achieving a cycle of “chemical softening—mechanical removal”. Experimental results show that an ultra-smooth surface with a surface roughness Ra of 0.0817 nm can be obtained under alkaline conditions (pH = 9), and the material removal rate is enhanced with different catalysts.

Electrochemical magnetorheological hybrid polishing (EC-MRF/H-ECMR) technology generates an oxide film on the workpiece surface through a controlled electrochemical reaction, which is then removed by the mechanical synergy of the magnetorheological polishing pad, thereby achieving high-efficiency and high-precision synergistic processing. Compared with conventional MRF, ECMRF can reduce surface roughness more rapidly and uniformly, and its polishing effect is particularly significant for difficult-to-machine materials such as SiC and GaN [120].

The advantage of electro/chemical-assisted MRF technology lies in its significant improvement of material removal efficiency, making ultra-precision machining of difficult-to-machine materials (such as single-crystal SiC, GaN, sapphire, etc.) possible. The limitations are: (1) precise control of the chemical reaction rate and uniformity is relatively difficult; (2) chemical reagents may cause environmental pollution, and the waste liquid treatment cost is high; (3) the equipment requires additional electric field or chemical fluid circulation systems, increasing structural complexity and cost.

#### 4.2.3. Laser-Assisted MRF

Laser-Assisted MRF (LAMRF) uses a laser beam to locally heat the MR polishing fluid, thereby optimizing the processing performance by changing the viscosity of the fluid. The study by Zhang et al. [121] revealed the unique advantages of LAMRF: in conventional MRF processing, when the working gap is very small, the high-viscosity MR polishing fluid may accumulate at the entrance of the polishing zone, forming a “fluid pile-up” effect that reduces shear force and polishing efficiency. By irradiating the MR polishing fluid at the entrance of the polishing zone with a laser, its viscosity can be locally reduced, allowing the fluid to enter the working gap more easily and avoiding fluid accumulation. Experimental results show that laser assistance slightly decreases the cutting force, but significantly improves the processing quality, reducing the surface roughness Ra from approximately 12 nm (without laser) to 7 nm.

Laser-assisted MRF is mainly suitable for precision polishing conditions that demand extremely high surface quality and involve very small working gaps. The equipment is relatively simple, requiring only the integration of a laser optical path system into the existing MRF setup. However, it is currently applicable mainly to ductile materials; the improvement effect on high-hardness brittle materials needs further validation. Moreover, laser heating may accelerate the volatilization or decomposition of certain chemical components in the polishing fluid, and the thermal impact during long-duration processing requires systematic evaluation.

## 5. Process Parameter Optimization and Dwell Time Algorithms

### 5.1. Synergistic Optimization of Process Parameters and Magnetic Field

#### 5.1.1. Influence of Key Process Parameters

The finishing performance of MRF is comprehensively influenced by a variety of process parameters, mainly including the working gap, magnetic induction intensity (current), rotational speed of the polishing wheel/workpiece, processing time, and abrasive parameters (particle size, concentration, type), among others [55,129,130,131,132,133]. Systematically analyzing the quantitative effects of these parameters on the material removal rate (MRR) and surface roughness is the fundamental prerequisite for achieving process optimization.

Working gap refers to the minimum distance between the polishing wheel and the workpiece surface, and its magnitude directly affects the magnetic induction intensity and hydrodynamic pressure. Yin et al. [134] conducted experiments with a large-size electromagnetically excited polishing tool and found that as the working gap increased from 1.0 mm to 1.8 mm, the surface roughness gradually increased (from 0.7 nm to about 3.0 nm). This is because a larger gap reduces the magnetic flux density at the workpiece surface; when the magnetorheological polishing fluid can no longer maintain sufficient hardness, the abrasive particles lose effective constraint, and the processing quality rapidly deteriorates. Fu et al. [135] systematically measured the variations of normal force and shear force under different polishing gaps (0.2–1.0 mm) based on magnetorheological hydrodynamic hybrid (MRHC) polishing. They found that the normal force decreased from 29.7 N to 24.5 N, and the shear force decreased from 1.9 N to 1.2 N, verifying the direct controlling effect of the gap on polishing forces.

The magnetic induction intensity (current) is the most sensitive parameter affecting the finishing performance of MRF. As the current increases, the magnetic field strength increases, the magnetic dipole interaction intensifies, the magnetic particle chains become denser and stronger, and the apparent viscosity and yield stress of the fluid increase accordingly, which macroscopically manifests as an increase in polishing pressure and material removal rate. However, this gain is not unlimited. When the current further increases to the point where the magnetic circuit of the iron core reaches magnetic saturation, the increase in magnetic induction intensity slows down, and the material removal rate correspondingly tends to stabilize [136]. Therefore, in equipment design, the magnetic saturation threshold of the iron core should be increased as much as possible to expand the adjustable range of the magnetic field strength.

The rotational speed includes both the workpiece spindle speed and the polishing tool speed. An increase in rotational speed, on the one hand, raises the relative linear velocity between the workpiece and the polishing film (corresponding to the V term in the Preston equation); on the other hand, it also enhances the hydrodynamic pressure effect in the polishing zone. However, excessively high rotational speeds may bring about the following adverse effects: (1) The centrifugal force becomes too large, causing the polishing fluid to be thrown out of the processing zone, thereby reducing the number of abrasive particles effectively involved in material removal. (2) Frictional heating intensifies, raising the temperature of the polishing fluid and decreasing its viscosity. Song et al. [131] compared four speeds—1120, 980, 840, and 700 rpm—under the same total number of revolutions. They found that while higher speeds can reduce processing time, excessively high speeds (e.g., 1120 rpm over extended operation) can degrade surface quality due to temperature rise and fluid splashing, ultimately causing Ra to increase. In contrast, a combination of high and low speeds (e.g., high speed followed by low speed) achieved an axial Ra of 0.04 μm within 1602 cycles, saving approximately 40% of the cycle count compared to a single fixed speed. This confirms the effectiveness of a variable-speed strategy in improving processing efficiency.

Processing time: In the initial stage of MRF processing, the rapid removal of surface asperities results in a relatively high material removal rate (MRR). As processing proceeds, the surface becomes smoother, and the MRR gradually decreases and eventually stabilizes. Song et al. [131] found that during VMRP polishing of titanium alloy tubes, the axial Ra tended to stabilize after 2025 cycles (decreasing from 0.23 μm to 0.079 μm); further extension to 2700 cycles only reduced it to 0.081 μm, while the circumferential Ra continued to decrease to 0.07 μm. This indicates that the axial roughness is primarily controlled by circumferential polishing marks, and increasing the processing time offers limited improvement in the axial direction. This “time saturation effect” dictates that the processing duration must be reasonably selected when establishing the machining process to avoid ineffective overprocessing.

The relative size of abrasive particles to magnetic particles is a key parameter for optimizing the magnetorheological finishing effect, requiring a trade-off between material removal capability and surface quality. Studies have shown that when the abrasive particle size is close to that of carbonyl iron powder (CIP) (e.g., both 6 μm), the MR fluid exhibits the highest yield stress and saturation magnetization, and the greatest improvement in surface roughness (approximately 65.85% reduction), demonstrating excellent polishing performance. In contrast, when the abrasive particles are either too large (9 μm) or too small (4 μm), the continuity of the CIP chains is disrupted, leading to a decrease in yield stress and a significant reduction in the surface roughness improvement. Therefore, in MRF process design, abrasives that match the size of the magnetic particles should be preferentially selected to achieve a good balance between material removal capability and processed surface quality [54]. In addition, the use of a multi-particle-size combination (rough polishing followed by fine polishing) can balance material removal efficiency and surface quality. Song et al. [131] sequentially applied a combination of CIP/diamond with particle sizes of 18/20 μm, 10/10 μm, and 5/5 μm, and after 2700 cycles, they optimized the axial and circumferential Ra values to 0.05 μm and 0.038 μm, respectively. This demonstrates that smaller particles can effectively eliminate the polishing marks left by larger particles, thereby realizing a process strategy that combines rough and fine polishing.

The influence trends of the above process parameters on MRR and surface roughness have been extensively studied; however, most studies focus on qualitative description of the trends, while quantitative analysis of parameter influence intensity and reports on process repeatability remain relatively limited. In recent years, research has begun to address this issue. Bi et al. [125] employed an orthogonal experimental method to investigate the contribution rates of ultrasonic power, polishing pad rotation speed, and abrasive concentration to MRR in ultrasonic-assisted magnetorheological polishing (UMRF). Their results showed that the contribution rate of ultrasonic power was as high as 57.9%, far exceeding those of polishing pad rotation speed (13.3%) and abrasive concentration (12.5%), quantitatively revealing the dominant role of ultrasonic vibration in the hybrid process. Regarding the repeatability of surface roughness, Yin et al. [134] conducted five sets of repeated polishing experiments at different working gaps (1.0–1.8 mm) and calculated the coefficient of variation (CV) of the measured Ra values, which ranged from 6.8% to 15.2%. The smallest gap (1.0 mm) yielded the lowest CV (6.8%), indicating that process repeatability is optimal when process parameters are optimized. Liang et al. [74] performed three independent repeated experiments under identical process parameters in chemical–magnetorheological finishing (CM-MRF) of single-crystal SiC. The standard deviation of MRR was ±12.5 nm/h, and the standard deviation of surface roughness was ±0.008 nm (with a mean Ra of 0.0817 nm), demonstrating good process stability.

It should be noted that many reported surface roughness values (e.g., Ra < 1 nm) are based on single measurements at a single sampling location using a white light interferometer or atomic force microscope. In practice, the measurement uncertainty of these instruments at the sub-nanometer scale is typically ±0.2 nm, and variations in sampling location and number of measurements can lead to significant fluctuations in the measured values. Therefore, when reporting MRF processing quality, it is advisable to also provide the standard deviation or coefficient of variation from repeated experiments to enable repeatability assessment and cross-laboratory comparison.

Moreover, most parametric studies report only main effects without investigating interaction effects among parameters (e.g., gap vs. magnetic field, rotational speed vs. fluid temperature). Such interactions can lead to non-intuitive optimal regions, and ignoring them may result in suboptimal process windows. Future research should employ design of experiments (DoE) methodologies, such as response surface or factorial designs, to systematically quantify these interactions and resolve contradictory findings in the literature.

To improve the reproducibility and cross-study comparability of MRF results, we strongly recommend that future experimental reports adhere to the following reporting standards: (1) clearly specify the measurement instrument and its calibration status; (2) report scan size and number of measurement points; (3) provide the arithmetic mean roughness (Ra) together with the standard deviation (SD) and coefficient of variation (CV); (4) state the sampling location and whether the reported value is an average over the full surface. For sub-nanometer roughness values, also mention the instrument’s noise floor and the resolution limit, as uncertainties of ±0.2 nm are common, which may outweigh the reported differences among different processes.

#### 5.1.2. Magnetic Field Configuration and Design Optimization

Magnetic field configuration is one of the core factors affecting the finishing efficiency and uniformity of MRF [84]. The generation methods of gradient magnetic fields can be divided into two major categories: permanent magnet excitation and electromagnetic excitation. The permanent magnet excitation features a simple structure, no need for cooling, and low cost, but its magnetic field strength is fixed and non-adjustable, and there is a risk of demagnetization over prolonged use. Electromagnetic excitation offers adjustable current, controllable magnetic field strength, and fast response, but requires a dedicated power supply and heat dissipation system, resulting in a larger equipment footprint. In practical applications, wheel-type and ball-end tools mostly adopt electromagnetic excitation, whereas disk-type and cluster tools mainly use permanent magnets.

The multi-pole magnetic field configuration and cluster effect involve arranging multiple magnetic poles in a specific geometric pattern to form a large-area, high-intensity gradient magnetic field region, which is a key technology for improving the processing efficiency of disk-type MRF. The arrangement of the magnetic poles directly affects the magnitude, uniformity, and direction of the magnetic flux density within the polishing zone. Nie et al. [84] systematically investigated the influence of four magnetic pole orientation arrangements (same-direction arrangement, cross-layer arrangement, cross-line arrangement, and cross-orientation arrangement) on the magnetic field distribution based on a multi-pole disk-type MRF tool. By introducing magnetic field characteristic parameters based on magnetic flux density—namely $B_{\mathrm{rms}}$, $B_{\mathrm{avg}}$, $B_{\mathrm{flat}}$, and $B_{\mathrm{fluc}}$—to comprehensively evaluate each scheme, the results showed that the cross-orientation arrangement exhibited the best performance in terms of magnetic flux density strength, flatness, and fluctuation. Furthermore, three magnetic pole arrangement patterns—checkerboard, circumferential, and honeycomb—were further analyzed, and it was found that the honeycomb arrangement, owing to its equidistant characteristics, could generate a more uniform magnetic field distribution. The validity of the model was verified experimentally, and it was noted that large-diameter magnetic poles are beneficial for improving polishing efficiency, whereas small-diameter magnetic poles help to improve workpiece form accuracy.

The optimization of magnetic pole shape also plays a significant role in improving the uniformity of magnetic flux density distribution in the processing zone. Bedi et al. [137] compared the effects of flat-plate-shaped and curved-plate-shaped permanent magnets on the MRF processing of cylindrical outer surfaces and found that the curved magnet generated a more uniform magnetic flux density distribution in the working gap, and its surface roughness improvement rate was 8.93% higher than that of the flat-plate magnet.

The Halbach array is a special magnet arrangement that concentrates magnetic field lines in a specific direction, thereby generating a significantly enhanced magnetic field on one side [138]. Xie et al. [139] applied an optimized Halbach circularly symmetric array to the excitation system of an MRF polishing disk. Compared with a non-circularly symmetric array, the magnetic field intensity was increased by approximately 33%, significantly improving the directional alignment of the abrasive particles and the processed surface quality. Experimental verification showed that this optimized array can achieve an ultra-smooth surface with a surface roughness Ra as low as 12 nm.

The greatest limiting factor for the optimization of magnetic field configuration is the three-dimensional spatial constraint. If the installation space for the magnetic poles is extremely narrow (e.g., in small-diameter ball-end tools), the optimal arrangement of the magnetic poles becomes very difficult. Disk-type tools with multi-pole excitation, owing to their higher spatial degrees of freedom, are the primary focus of magnetic field optimization research. For tools with single-pole excitation, only limited improvement of the magnetic field distribution can be achieved by changing the shape of the magnetic pole.

### 5.2. Dwell Time Algorithms and Path Planning

The dwell time algorithm is a core technology for achieving precise correction of workpiece form errors in CCOS deterministic polishing. Its fundamental problem is: given the initial form error distribution of the workpiece and the tool influence function (TIF) of the polishing tool, to determine the optimal dwell time of the polishing tool at each point on the workpiece surface such that the form error is maximally removed [140,141,142].

The solution for dwell time is essentially a deconvolution optimization problem. Existing solution methods can be broadly classified into the following categories:

(1) Direct matrix-based solution methods: The form error distribution, tool influence function, and dwell time are discretized into matrix forms, transforming the deconvolution problem into solving a system of linear equations. Li et al. [24] combined the non-negative least squares method with adaptive regularization to propose a fast dwell time algorithm based on a matrix computation model, effectively addressing the computational efficiency issue in high-precision MRF processing of large-aperture optical components. Zhang et al. [142] converted the deconvolution operation of dwell time into matrix operations and solved the optimization model using least-squares approximation and best uniform approximation.

(2) Frequency-domain method based on Fourier transform: The Fourier transform is used to convert the deconvolution problem in the time domain into a division operation in the frequency domain, and the dwell time distribution is obtained by inverse transformation. This method is simple in principle and requires low computational effort, but it cannot easily handle the physical constraint that dwell times must be non-negative.

(3) Iterative optimization algorithms: An iterative scheme is constructed to gradually approach the optimal dwell time solution. Zhang et al. [142] proposed a fast iterative algorithm based on sparse matrices, which can obtain a smooth, continuous, and non-negative dwell time distribution while satisfying the dynamic characteristics of the computer numerical control system.

(4) Modern heuristic algorithms: In recent years, heuristic algorithms such as genetic algorithms and particle swarm optimization have also been introduced for dwell time solution. Gao et al. [143] proposed a particle swarm optimization dwell time algorithm, which obtains the optimal dwell time value at each dwell point through a comprehensive evaluation of the overall dwell time, providing a new approach for dwell time optimization.

Path planning: The two most commonly used trajectories in MRF processing are the raster path and the spiral path [144]. The raster path scans the entire workpiece surface through equally spaced parallel lines; the algorithm is simple and has strong adaptability, but it may introduce periodic mid-spatial frequency errors. The spiral path starts from the workpiece center and expands outward along a spiral; the motion is continuous and smooth, which can effectively reduce the generation of mid-spatial frequency errors, but its adaptability to non-circularly symmetric freeform surfaces is relatively poor. For the processing of large-aperture optical components, Qian et al. [145] established a chord-height error model for adjacent polishing points based on the raster trajectory and analyzed the influence of chord-height error on processing quality. Huang et al. [146] proposed a polishing trajectory interpolation method based on proportional feed rate adjustment, which significantly improved the dwell time solution accuracy and the convergence rate of form errors.

The edge effect is a common technical challenge in MRF processing, referring to the non-uniform material removal at the edge region of the workpiece caused by the change in contact state between the polishing film and the workpiece. Dong et al. [147] proposed a modified dwell time optimization model using iterative and numerical methods, which can obtain a smooth, continuous, and non-negative dwell time distribution map, achieving an RMS convergence efficiency as high as 99.6% and effectively suppressing the edge effect.

In summary, research on dwell time algorithms and path planning is evolving from simple surface shapes to complex freeform surfaces, from off-line static calculation to on-line adaptive adjustment considering dynamic characteristics, and has incorporated engineering practice factors such as edge effect suppression.

## 6. Applications and Challenges of Magnetorheological Finishing

### 6.1. Typical Applications

MRF technology, owing to its unique advantages of high precision, low damage, and strong controllability, has achieved important applications in multiple high-tech fields such as aerospace, biomedicine, optoelectronic information, and semiconductors.

The aerospace and defense sectors demand extremely high precision and reliability of optical components. As a core component of aerospace inertial navigation systems, the inner surface roughness of key parts of a hemispherical resonant gyroscope directly affects navigation accuracy. Using MRF for precision machining of spherical resonators can yield ultra-smooth curved surfaces with a surface roughness Ra as low as 3.2 nm [148]. In the processing of large-aperture optical mirrors, Li et al. [24] used a self-developed MRF device to perform magnetorheological finishing on a 1.5 m off-axis aspherical SiC mirror for a total of approximately 142 h. The surface form error PV value rapidly converged from an initial 1820.6 nm to 394.1 nm. Repeated processing verification experiments showed that the PV value deviation of the surface form after three independent processing runs was within ±28 nm, indicating good convergence stability. Guo et al. [149] adopted a magnetorheological elastomer-based Chemical Mechanical Polishing method to achieve ultra-smooth surface processing of SiC optical elements. Under optimized conditions, the surface roughness Ra decreased from 1.688 μm to 0.267 μm, and the material removal rate was 3.842 μg/h.

In the biomedical field, there are stringent requirements for the surface quality of implants and medical devices. Cobalt-chromium-molybdenum (CoCrMo) alloy is an important material for manufacturing artificial hip joints. After MRF processing, the surface roughness of CoCrMo alloy can reach 5 nm, significantly improving the wear resistance and biocompatibility of the joint [150]. Meanwhile, Rajput et al. [151] applied hybrid electrochemical magnetorheological (H-ECMR) polishing technology to improve the surface quality of bone plates fabricated by laser powder bed fusion (selective laser melting, SLM). After polishing, the surface roughness was significantly improved with minimal impact on the original geometrical morphology of the bone plates, demonstrating the application potential of MRF technology in the post-processing of additively manufactured biomedical implants. For ultra-high molecular weight polyethylene (UHMWPE) acetabular cups, MRF polishing increases the surface microhardness and correspondingly extends their service life [152]. Furthermore, MRF technology has also shown great application potential in the surface finishing of medical devices such as stainless steel 316 L implants and nickel–titanium shape memory alloy stents.

In the optoelectronic information and semiconductor fields, there are stringent requirements for the surface flatness and lattice integrity of substrate wafers. Using EC-MRF technology for ultra-smooth planarization of gallium nitride (GaN) wafers, a damage-free surface with Ra = 0.6 nm can be obtained in 180 min, and a surface with Ra = 0.9 nm in 60 min [120]. Furthermore, MRF technology has also been successfully applied to the precision processing of optoelectronic devices such as sapphire substrates, silicon carbide substrates, gallium arsenide wafers, and aspheric lenses in smartphone camera lens modules.

In the field of civil industry, the application of MRF technology is increasingly expanding into precision molds, high-end bearings, printing machinery, and other areas. After processing the inner surface of the outer ring of a ball bearing using rotary magnetorheological honing (RMRH), the surface finish can reach 0.06 μm, and the roundness and waviness are significantly improved [153]. For a textile grooved drum processed by MRF, the surface roughness Ra is reduced to 10 nm, which greatly reduces the yarn breakage rate [154].

The above application cases indicate that MRF technology has become an important precision machining method supporting high-end equipment manufacturing and advancing the development of frontier science and technology, with broad application prospects.

### 6.2. Current Technical Bottlenecks

Despite the significant progress made in MRF technology in recent decades, it still faces several technical challenges in large-scale industrial applications and for a broader range of material systems.

The trade-off between processing efficiency and accuracy is one of the core problems confronting MRF technology. The fundamental physical process of MRF involves the micro-scratching of the workpiece surface by micron-/nanometer-sized abrasive particles, which determines that MRF is inherently a low-material-removal-rate (MRR) process. Although multi-energy field hybrid techniques such as UA-MRF and EC-MRF can improve the MRR to some extent, the increased complexity and cost of the equipment severely limit their applicability. How to substantially increase the MRR while maintaining nanometer-scale processing accuracy remains a long-standing research topic in MRF technology.

Suppression of the edge effect: When the polishing film travels to the edge of the workpiece, the abrupt change in the support condition leads to an uneven distribution of contact pressure, causing an abnormal increase in material removal at the edge region and forming a “roll-off” effect, which severely affects the processing accuracy. The study by Hu et al. [155] indicates that the edge effect originates from the variation in the hydrodynamic pressure distribution of the magnetorheological polishing fluid at the workpiece boundary, and is essentially caused by the spatial abrupt change in the contact state between the polishing film and the workpiece. The main suppression methods currently available include the use of a small-scale removal function in the edge region and the compensation and correction of the removal function through dwell time algorithms. Both methods can effectively mitigate the edge effect and can be flexibly selected according to the actual processing conditions.

Path planning and accuracy control in aspheric/freeform surface machining constitute another technical challenge. For aspheric or freeform workpieces, due to curvature variations and continuous changes in the surface normal vector direction, the conventional CCOS method based on a constant tool influence function is no longer applicable. Although regionalized influence function modeling and five-axis linkage posture control can theoretically address this issue, their high computational complexity and stringent requirements on the dynamic characteristics of the machine tool make it difficult to simultaneously guarantee both accuracy and efficiency in practical applications.

The long-term stability and recycling service life of MR polishing fluid performance directly determine the economy and continuity of the MRF process. During prolonged continuous processing, MR polishing fluid may face the following changes: (1) volatilization of water or carrier liquid, leading to changes in component proportions; (2) wear failure of abrasive particles; (3) gradual consumption of chemically active components; (4) surface oxidation and magnetic performance degradation of magnetic particles. All these changes cause drift in the rheological characteristics and removal capability of the MR polishing fluid, thereby affecting processing stability and product consistency. How to develop polishing fluids with high stability and long service life and how to achieve online real-time monitoring and adaptive compensation of polishing fluid performance are important engineering problems facing the practical application of MRF technology.

The magnetic field distortion problem in machining magnetic materials is also a special challenge for MRF technology. Because ferromagnetic workpieces (e.g., carbon steel, martensitic stainless steel, nickel-based alloys) have high magnetic permeability, when placed in the processing magnetic field, they disturb the original spatial magnetic field distribution, leading to uneven film formation of the polishing fluid, local accumulation of abrasives, and deterioration of processing quality. To address this problem, current research directions include developing MR polishing fluid formulations that can weaken the magnetic response of the workpiece, optimizing the magnetic circuit design to minimize the influence of the workpiece on the magnetic field, and pre-coating a non-magnetic isolation layer on the workpiece surface.

## 7. Future Development Trends and Prospects

In response to the current technical bottlenecks and the ever-increasing application demands, the future development of MRF technology will revolve around four main directions: intelligentization, greenization, standardization, and deepening of fundamental theories.

### 7.1. Intelligent MRF Technology

With the rapid development of industrial and intelligent manufacturing technologies, integrating advanced sensing, artificial intelligence, and big data technologies into the MRF process is an inevitable trend in the development of MRF technology [156].

In-line inspection and real-time feedback control are the foundation of intelligentization. Integrating miniaturized sensors (such as micro force sensors, temperature sensors, eddy current sensors, etc.) into the MRF processing chamber enables real-time monitoring of the working gap, polishing pressure, and the temperature and viscosity of the polishing fluid. The focused-sensor-based in situ measurement method for ball-end MRF proposed by Iqbal et al. [157] allows real-time measurement of changes in the workpiece surface topography during processing, eliminating the repeated clamping errors and time costs associated with conventional off-line measurement.

Adaptive closed-loop control systems can dynamically adjust processing parameters based on online inspection data, keeping the process in an optimal state at all times. Zhang et al. [156] developed a six-degree-of-freedom robot-based MRF platform with an active force feedback control system, achieving high-stability and high-precision polishing of complex curved workpiece surfaces. By sensing changes in polishing force in real time and feeding back to adjust the tool posture, this system achieves significant improvements in form accuracy retention and surface quality consistency compared with traditional open-loop control systems.

Machine learning-assisted process modeling and optimization can significantly reduce experimental costs. Machine learning methods, including artificial neural networks (ANN), support vector regression (SVR), and deep learning, have been attempted for modeling the complex nonlinear mapping relationship between MRF process parameters and finishing outcomes [158,159]. Liang et al. [160] successfully predicted the variations of material removal rate (MRR) and surface roughness (Ra) with various process parameters in magnetorheological finishing using a backpropagation (BP) neural network model. The mean absolute percentage error of the model predictions was below 2%, validating the high-precision potential of machine learning methods for modeling complex polishing processes. In the future, approaches combining physical models with data-driven models are expected to achieve higher-precision process prediction models with fewer experimental samples.

### 7.2. Sustainable Manufacturing and Green Media

Green Manufacturing is the main theme of manufacturing development in the 21st century. The development of environmentally friendly, low-toxicity, and recyclable MR polishing fluids is crucial for promoting the sustainable development of MRF technology [6].

Researchers have developed a low-cost magnetorheological (MR) polishing fluid using synthetic oil as the carrier liquid, electrolytic iron powder as the magnetic particles, and xanthan gum as a green additive. The xanthan gum in this MR fluid is environmentally friendly and low-cost, can effectively improve the sedimentation stability of the particles, and has little effect on the saturation magnetization and coercivity of the iron powder. The prepared MR fluid is expected to achieve a yield stress exceeding 93.7 kPa, which is comparable to the performance of conventional carbonyl iron powder-based MR fluids, thereby significantly reducing the cost while maintaining a good magnetorheological effect [161]. The guar gum-borax hydrogel-based polishing fluid developed by Wei et al. [162] exhibits a unique shear-thickening characteristic, i.e., its viscosity increases with increasing shear rate. This property is beneficial for enhancing the clamping force on abrasives and the material removal efficiency during processing.

Polishing fluids made from renewable materials also represent an important approach to reducing environmental impact. Using waste tire rubber powder, recycled plastics, and other materials as carrier components of the MRF medium reduces waste emissions while lowering production costs, aligning with the concept of a circular economy.

Water-based carrier liquids are becoming a trend as substitutes for oil-based ones. Compared with traditional mineral oil-based and silicone oil-based MR polishing fluids, water-based polishing fluids offer significant advantages in terms of biodegradability, production cost, and workplace safety. The development focus of novel water-based systems lies in adding environmentally friendly rust inhibitors and antioxidants to address the key challenge of corrosion of metallic magnetic particles by the water-based medium.

The development of environmentally friendly MR polishing fluids using water-based carriers and natural additives represents a key direction for sustainable manufacturing. A notable example is the work by Milde et al. [51], who formulated a high-performance water-based MR polishing slurry using ferrofluid as the carrier liquid and sepiolite—a naturally abundant, low-toxicity, and biodegradable clay mineral—as the thickening agent. The addition of sepiolite not only reduced the sedimentation rate by 75% but also maintained a strong MR effect. Importantly, the use of water-based ferrofluid and natural clay avoids the environmental and health hazards associated with oil-based carriers and organic solvents commonly used in polymer coating synthesis. This study demonstrates that superior polishing performance and environmental sustainability can be achieved simultaneously, providing a viable pathway toward green MRF technology for industrial applications.

### 7.3. Standardization and Generalization of Process Equipment

For MRF technology to move from the laboratory to large-scale industrial applications, the current customization problem of “one machine for one use, a dedicated machine for a dedicated task” needs to be addressed.

Equipment standardization mainly includes: modular design of polishing tools (facilitating quick replacement and maintenance), unified control interface protocols, and streamlined processing technology packages. The commercial equipment mentioned above, such as the QED company’s Q series and the domestic MRP series, DQ series, and ZJY-NCM series, have already taken important steps toward standardization.

Generalization refers to integrating the MRF polishing unit with general-purpose multi-axis CNC machine tools or industrial robots, thereby lowering the high cost barrier associated with equipment specificity.

The establishment of process databases and processing standard specifications facilitates the rapid transfer of processing parameters and the sharing of experience among different manufacturers and equipment models, thereby avoiding a large amount of repetitive trial-and-error work.

## 8. Conclusions

After nearly half a century of development, magnetorheological finishing (MRF) technology has evolved from a laboratory concept to one of the key processes in the field of high-end precision manufacturing. This paper systematically reviews MRF technology from six dimensions: polishing fluid, removal mechanism, equipment and processes, hybrid technologies, process optimization, and typical applications. MR polishing fluid is composed of a carrier liquid, magnetic particles, abrasives, and additives. The Herschel–Bulkley model can well describe its nonlinear rheological behavior over a wide shear rate range. Current research focuses on improving sedimentation stability through surface modification, nanoparticle addition, and core–shell structure design, as well as developing environmentally friendly carrier liquids such as water-based and ionic liquid-based fluids. Material removal is the result of the synergistic action of mechanical scratching, chemical corrosion, and rheological extrusion, among which shear stress is more critical than normal pressure. Atomic-scale molecular dynamics simulations have revealed the microscopic mechanism that chemical reactions can weaken surface atomic bonding and lower the threshold for mechanical removal. In terms of equipment, the three main configurations—wheel-type, ball-end-type, and disk-type/cluster-type—are respectively suitable for large-aperture aspheric surfaces, small-curvature freeform surfaces, and high-efficiency planar processing. In recent years, the rapid development of the magnetorheological jet polishing (MJP) configuration has further enriched the equipment family of MRF, making it the fourth typical configuration after wheel-type, ball-end-type, and disk-type. MJP leverages the synergistic effect of high-pressure jet flow and the magnetic field at the nozzle outlet to achieve non-contact, tool-wear-free flexible polishing, and is insensitive to working distance. It is particularly suitable for deterministic figuring and deburring of steep concave surfaces, micro-cavities, and functional microstructured surfaces (e.g., micropyramid arrays, microlens arrays, microfluidic chips, etc.) [163]. Multi-energy field hybrid assistance technologies such as ultrasound, electrochemistry, and laser significantly enhance the processing efficiency and surface quality of hard and brittle materials. Among the key process parameters, the working gap, magnetic induction intensity, and relative velocity have the most significant effects on material removal rate and surface roughness. Proper matching of abrasive and magnetic particle sizes, optimized magnetic pole arrangements (e.g., cross-orientation arrangement and Halbach array), and improvements in dwell time algorithms are core aspects for ensuring processing accuracy and uniformity. At present, MRF technology has found typical applications in aerospace (large-aperture SiC mirrors), biomedical (artificial joints), and semiconductor (GaN wafers) fields. Nevertheless, challenges remain, including the trade-off between processing efficiency and accuracy, edge effects, polishing fluid service life, and magnetic field distortion when machining magnetic materials. It is worth noting that although MJP is relatively limited in terms of processing efficiency, its insensitivity to working distance and its freedom from complex tool-path compensation make it irreplaceable for polishing complex internal cavities and microstructures. This compensates for the shortcomings of wheel-type and ball-end-type tools in terms of spatial accessibility, representing an indispensable and important direction for the diversified development of MRF technology [163].

In the future, MRF technology will develop toward intelligent, green, standardized, and generalized solutions, as well as multi-scale collaborative simulation. It is expected to replace traditional polishing methods on a broader scale and become a mainstream technology for next-generation ultra-precision machining.

## Figures and Tables

**Table 1:** Comparison with previous reviews.

Review	Scope	Classification Method	Intelligentization Coverage	Process Modeling Discussion
Sidpara (2014) [35]	Polishing fluid and forces	Not classified	None	Empirical Preston-based
Bedi & Singh (2016) [34]	Tool types (general)	By surface shape	None	Limited
Xiao et al. (2016) [36]	Hybrid technologies	By auxiliary energy	None	None
Duy et al. (2026) [6]	Process parameters	By material	Brief mention	Shear stress models
This review	Full chain (fluid, mechanism, tools, hybrid, intelligence)	By tool morphology and energy field	ML-based modeling, in situ sensing, adaptive control	Multi-physics (hydrodynamic, granular flow, MD/ReaxFF)

**Table 2:** Comparison of characteristics of several nonlinear rheological models [38].

Model	Features	Drawbacks	Applicability
Bingham plastic model	Fluids only flows when surpasses the critical shear stress	The initial part of the shear stress–strain rate curve cannot be described. Cannot capture shear thinning effect	Suitable for wide range of strain rate application scenarios
Herschel–Bulkley model	Describes shear-thinning or shear-thickening fluids with yield stresses in high strain rate	Parameters need to be obtained by fitting experimental data. However, the estimation of parameters is relatively complex	Suitable for predicting rheological behavior under extreme conditions
Casson model	Some structural changes, such as viscoelasticity, that occur at lower shear rates can be captured	The prediction ability is weak at high shear rate	Suitable for capturing nonlinear behavior at low strain rate
Power law	Multiple nonlinear processes can be described simultaneously	Purely empirical equation, physical meaning is not clear. Only applicable to moderate strain rate	Suitable for scenes that emphasize shear thinning behavior
Biviscosity model	The fluid velocity and stress fields can be determined when below the yield stress	Just an empirical model, relying on experimental data and the parameter numbers is relatively large and complicated	Suitable for highly nonlinear MR fluids

**Table 3:** Types of workpieces suitable for various polishing tools.

Types	Plane	Large-Curvature Curved Surface	Small-Curvature Curved Surface	Side Surface	Integrally Immersed Type
Disk Type (Grooved)	√				
Wheel Type	√	√			
Ball-End Type	√	√	√		
Cylindrical Type	√			√	
Fully Immersed Type	√				√

**Table 4:** Advantages and disadvantages of typical MRF tool configurations.

Polishing Method	Advantages	Disadvantages	Typical Applications
Wheel-type MRF	Most mature technology, highest commercialization level; integrated with CCOS for deterministic finishing; suitable for large-aperture, aspherical, and freeform optical components	Point contact leads to low material removal rate (MRR); prone to mid-spatial frequency errors; expensive equipment; workpiece aperture limited by wheel size (≥8 mm)	Large optical mirrors, aspheric lenses
Ball-end MRF	High flexibility, suitable for large-curvature radii and complex freeform surfaces (e.g., artificial joints, molds); can machine small apertures and irregular shapes	Point contact results in low removal efficiency, not suitable for mass production; requires high-precision displacement control, increasing cost	Knee joint implants, small optical components with high curvature
Disk/Cluster type MRF	Surface contact provides large finishing area and high removal efficiency; dynamic magnetic field improves self-repair capability and uniformity; ideal for planar optics flattening	Only suitable for flat or near-flat workpieces; difficulty in fluid circulation affects stability; complex magnet arrangement design	Large flat wafers (sapphire, SiC), optical flats
Rotational MRF	Suitable for simultaneous finishing of blind hole inner walls and bottom surfaces	Poor versatility, limited to small-size blind holes	Blind holes, inner bores
Belt-type MRF	Large contact area, high MRR (up to 5× that of conventional methods)	Single finishing direction, prone to residual striae, limiting surface quality	Large planar optics (roughing stage)
Reciprocating MRF	Simple structure, low cost, can eliminate finishing textures	Low MRR, applicable materials limited (mainly optical glass)	Small optical samples, lab-scale polishing

**Table 5:** Advantages and disadvantages of hybrid MRF technologies.

Hybrid Technology	Advantages	Disadvantages	Typical Materials
Ultrasonic-assisted MRF (UA-MRF)	Increases MRR (up to 3.4×), improves surface quality; suitable for hard-brittle materials like SiC and sapphire	Difficult to apply to large-volume tools; mainly suited for small-to-medium sized workpieces	Sapphire, SiC, titanium alloy
Electrochemical-assisted MRF (EC-MRF)	Enables ultra-smooth, damage-free finishing of difficult-to-machine materials	Complex control of chemical reactions; high equipment cost; risk of waste pollution	GaN, SiC, wide-bandgap semiconductors
Chemo-mechanical MRF (CM-MRF)	Increases removal efficiency, suitable for high-hardness materials	High consumption of chemical reagents; difficulty in controlling reaction uniformity	Single-crystal SiC, quartz glass
Laser-assisted MRF (LAMRF)	Reduces polishing fluid viscosity, improves flowability; surface roughness Ra reduced from 12 nm to 7 nm	Mainly applicable to plastic materials; thermal effects need evaluation	Ductile metals, glasses

## Data Availability

No new data were created or analyzed in this study. Data sharing is not applicable to this article.
